# Chloroquine reverses chemoresistance via upregulation of p21^WAF1/CIP1^ and autophagy inhibition in ovarian cancer

**DOI:** 10.1038/s41419-020-03242-x

**Published:** 2020-12-04

**Authors:** Jae Ryoung Hwang, Woo Young Kim, Young-Jae Cho, Ji-Yoon Ryu, Jung-Joo Choi, Soo Young Jeong, Myeong-Sun Kim, Ji Hye Kim, E. Sun Paik, Yoo-Young Lee, Hee-Dong Han, Jeong-Won Lee

**Affiliations:** 1 Research Institute for Future Medicine, Samsung Medical Center, Sungkyunkwan University School of Medicine, Seoul, Republic of Korea; 2 Department of Obstetrics & Gynecology, Kangbuk Samsung Hospital, Sungkyunkwan University School of Medicine, Seoul, Republic of Korea; 3 Department of Obstetrics and Gynecology, Samsung Medical Center, Sungkyunkwan University School of Medicine, Seoul, Republic of Korea; 4 Department of Obstetrics and Gynecology, Dankook University College of Medicine, Cheonan, Chungnam Republic of Korea; 5 Department of Obstetrics and Gynecology, Kangbuk Samsung Hospital, Sungkyunkwan University School of Medicine, Seoul, Republic of Korea; 6 Department of Immunology, School of Medicine, Konkuk University, Chungju, Republic of Korea; 7 Institute for Refractory Cancer Research, Samsung Medical Center, Seoul, Republic of Korea; 8 Samsung Advanced Institute for Health Sciences & Technology, Sungkyunkwan University School of Medicine, Seoul, Republic of Korea

**Keywords:** Cancer therapeutic resistance, Ovarian cancer, Translational research

## Abstract

Overcoming drug-resistance is a big challenge to improve the survival of patients with epithelial ovarian cancer (EOC). In this study, we investigated the effect of chloroquine (CQ) and its combination with cisplatin (CDDP) in drug-resistant EOC cells. We used the three EOC cell lines CDDP-resistant A2780-CP20, RMG-1 cells, and CDDP-sensitive A2780 cells. The CQ-CDDP combination significantly decreased cell proliferation and increased apoptosis in all cell lines. The combination induced expression of γH2AX, a DNA damage marker protein, and induced G2/M cell cycle arrest. Although the CQ-CDDP combination decreased protein expression of ATM and ATR, phosphorylation of ATM was increased and expression of p21^WAF1/CIP1^ was also increased in CQ-CDDP-treated cells. Knockdown of p21^WAF1/CIP1^ by shRNA reduced the expression of γH2AX and phosphorylated ATM and inhibited caspase-3 activity but induced ATM protein expression. Knockdown of p21^WAF1/CIP1^ partly inhibited CQ-CDDP-induced G2/M arrest, demonstrating that knockdown of p21^WAF1/CIP1^ overcame the cytotoxic effect of the CQ-CDDP combination. Ectopic expression of p21^WAF1/CIP1^ in CDDP-treated ATG5-shRNA/A2780-CP20 cells increased expression of γH2AX and caspase-3 activity, demonstrating increased DNA damage and cell death. The inhibition of autophagy by ATG5-shRNA demonstrated similar results upon CDDP treatment, except p21^WAF1/CIP1^ expression. In an in vivo efficacy study, the CQ-CDDP combination significantly decreased tumor weight and increased expression of γH2AX and p21^WAF1/CIP1^ in A2780-CP20 orthotopic xenografts and a drug-resistant patient-derived xenograft model of EOC compared with controls. These results demonstrated that CQ increases cytotoxicity in combination with CDDP by inducing lethal DNA damage by induction of p21^WAF1/CIP1^ expression and autophagy inhibition in CDDP-resistant EOC.

## Facts

- Overcoming drug-resistance is a major challenge in improving the survival of patients with epithelial ovarian cancer (EOC).
- Chloroquine is an anti-malaria drug and inhibits autophagy.
- Chloroquine in combination with cisplatin increases cytotoxicity of drug-resistant EOC through induction of lethal DNA damage.
- Induction of p21^WAF1/CIP1^ expression and inhibition of autophagy mediated by chloroquine lead to lethal DNA damage.
- Combination of chloroquine with cisplatin represents a novel strategy to treat drug-resistant EOC.

## Introduction

Platinum-based drugs are generally accepted main drugs for treatment of epithelial ovarian cancer (EOC). However, some patients with EOC have intrinsic resistance or develop acquired resistance due to repeated administration^1,2^. Overcoming drug resistance is thus a big challenge to improve the survival of EOC patients^3,4^. Chemotherapeutic drugs for EOC such as platinum-based cisplatin (CDDP) are known to cause DNA damage, and leads to cell death^5,6^. However, chemo-resistant cancer cells can survive from anti-cancer drug-induced DNA damage via DNA repair pathways or bypassing cell cycle checkpoints^7,8^. Therefore, DNA damage/repair-related signaling pathways are important targets in treatment of chemo-resistant cancer.

Protein kinases including ataxia telangiectasia mutated (ATM) and RAD3-related (ATR) recognize DNA damage and induce DNA damage response signals^9,10^. Upon DNA damage, ATM/ATR activate the checkpoint kinases Chk2 and Chk1 by phosphorylation and also activate p53, inducing p21^WAF1/CIP1^ (also called Cyclin-dependent kinase inhibitor 1) and inhibiting Cyclin-dependent kinases to arrest the cell cycle and repair DNA damage. ATM/ATR phosphorylate histone variant H2AX at serine 139 which is called γH2AX in the response of DNA damage and therefore γH2AX is considered as a DNA damage marker protein^11^. Cyclin-dependent kinase 1, Cdk1 (Cdc2), is involved in cell cycle progression through an interaction with cyclin B1. Phosphorylation of Cdc2 at tyrosine 15 (Cdc2-pY15), an inactive form, arrests the cell cycle at G2/M^12^. During cell cycle arrest, DNA can be repaired, allowing cell survival, or persistent DNA damage induces cell death. In DNA-damaging anti-cancer agents, repair and cell cycle checkpoints are considered as mechanisms for acquiring resistance^13^.

Autophagy can be a survival signal under hypoxic and limited nutrient conditions, which are hallmarks of cancers^14,15^. Drug-resistant cancers can survive through induction of autophagy by anti-cancer drugs; therefore, autophagy was considered to induce resistance in chemotherapy. Therefore, autophagy inhibitors have become candidates to treat drug-resistant cancers.

Drug-resistant cancers can survive through induction of autophagy^14,15^; therefore, autophagy was considered to induce resistance in chemotherapy. Therefore, autophagy inhibitors have become candidates to treat drug-resistant cancers.

Chloroquine (CQ) is an anti-malaria drug and inhibits autophagy through inhibition of autophagosome-lysosome fusion^16^. The anti-tumor effect of CQ has been reported in different types of cancer^17,18^. CQ induced cell death through activation of p53 pathway in glioblastoma^19^. Cancer cells can survive from oxidative stress by removing reactive oxygen species (ROS) by autophagy. CQ induced cancer cell death by inhibiting autophagy-mediated removal of ROS^20^. CQ was also studied as an anti-cancer drug in combination with other anti-cancer drugs. CDDP induced cell proliferation through activation of the Akt/mTOR signaling pathway by activation of autophagy, and CQ induced cell death by inhibition of CDDP-induced autophagy in ovarian cancer cell lines^21^. CQ combined with an mTOR inhibitor, enhanced radiosensitivity of colon cancer^22^. Although there are several results on the cytotoxic effect of combined CQ and anti-cancer drugs, the exact functional mechanism or targeting signals have not been identified.

p21^WAF1/CIP1^ is induced by p53 upon DNA damage and has critical roles in cell cycle distribution, apoptosis, cell growth, and senescence^23^. The function of p21^WAF1/CIP1^ in cancer cell growth has been reported that ectopic expression of p21^WAF1/CIP1^ induced apoptosis in several cancers^24–26^. Furthermore, anti-cancer drugs induced cell death by induction of p21^WAF1/CIP1^ expression^27,28^. In contrast, the anti-apoptotic effect of p21^WAF1/CIP1^ in response of anti-cancer drugs has been reported in prostate and colon cancers^29,30^. Conflicting function of p21^WAF1/CIP1^ on cell growth and apoptosis is dependent on subcellular localization, post-translational modifications, and p53 status^31^. p21^WAF1/CIP1^ inhibited Cdc2 and cyclin B1 expression, thereby induced G2/M arrest and also induced S phase arrest by inhibition of Cdk2^31^. p21^WAF1/CIP1^ interacts with many proteins such as PCNA, Cdk2, and Cdk4, so the function of p21^WAF1/CIP1^ might be dependent on the cell context^27,32^.

In this study, we evaluated the effect of CQ on DNA damage/cell cycle in regard to cytotoxicity in drug-resistant EOC. We found that the CQ-CDDP combination increased cytotoxicity in drug-resistant EOC cell lines and induced γH2AX, demonstrating that the induced cytotoxicity might be caused by severe DNA damage. The CQ-CDDP combination sustained G2/M arrest and induced expression of p21^WAF1/CIP1^. The induced cytotoxicity of EOC by CQ was dependent on its autophagy inhibitor activity and induction of p21^WAF1/CIP1^. In an in vivo study, the CQ-CDDP combination significantly decreased tumor weights in drug-resistant cell line-derived xenograft and patient-derived xenograft (PDX) models. Taken together, these results demonstrated that CQ could increase cytotoxicity in combination with CDDP by inducing lethal DNA damage through induction of p21^WAF1/CIP1^ expression and autophagy inhibition in CDDP-resistant EOC.

## Materials and methods

### Cell lines

Ovarian carcinoma cell lines A2780 (cisplatin-sensitive) and A2780-CP20 (cisplatin-resistant) were obtained from American Type Culture Collection (ATCC) and Dr. Anil K. Sood (M&D Anderson Cancer Center, Houston, TX, USA), respectively. Cells were maintained in RPMI (Gibco, CA, USA) containing 10% FBS and 100 units/ml penicillin/100 µg/ml streptomycin (Invitrogen, CA, USA) and were grown at 37 °C in a 5% CO_2_ incubator. RMG-1, an ovarian clear cell carcinoma cell line, was purchased from Japan Health Science Research Resources Bank (HSRRB, Osaka, JAPAN) and maintained in HAM’s F-12 media (Gibco) containing 10% FBS and penicillin/streptomycin. We obtained the authentications of the cell lines used in this study and checked the certificates of the cell lines using species verification and short tandem repeat DNA profiling assay. CDDP was purchased from Sigma (MO, USA) and suspended in distilled water. CQ purchased from Sigma was suspended in distilled water at 50 mg/ml concentration and used at 10–30 µM in all cell lines. Inhibitors for ATM (KU55933, Abcam, England) and ATR (AZD6738, AstraZeneca, England) were used at 15 µM and 1 µM, respectively. MG-132 (Z-L-Leu-D-Leu-L-Leu-al) and bafilomycin A1 were purchased from Sigma and used at 2 µM and 0.1 µM, respectively.

### Preparation of lentivirus-expressing shRNA and stable cell lines

To prepare lentiviruses expressing shRNA, plasmids for lentivirus preparation, pMD.2G (Addgene, MA, USA) and psPAX2 (Addgene), and plasmid encoding either ATG5-, p21^WAF1/CIP1^-specific shRNA (Origene, MD, USA), or GFP-expressing scrambled shRNA (Origene) were transfected into 293T cells using Lipofectamine 2000 (Invitrogen). Lentivirus expressing shRNA was prepared as previously described^33^. Virus-containing media were stored in aliquots at −80 ^o^C.

For preparing stable cells, A2780-CP20 cells were plated into 60 mm dishes one day before lentivirus infection. Cells were infected with lentivirus expressing ATG5-specific shRNA, p21^WAF1/CIP1^-specific shRNA or scrambled control shRNA in the presence of polybrene (8 µg/ml, Sigma) for 6 h and media was changed to complete growth media (RPMI media with 10% FBS and penicillin/streptomycin). At 48 h after virus infection, media were changed to complete growth media containing puromycin (Sigma). Cultures were monitored for surviving cells and surviving cells were either used for experiments or stored in RPMI media containing 20% FBS and 10% DMSO (Sigma) at −80 ^o^C.

### Western blot analysis

EOC cells were plated at 2 × 10^5^ cells/well in a 6-well plate. Cells were treated with CDDP, CQ, or CQ-CDDP combination at the indicated concentrations for 24 h or 48 h. Cells were lysed in RIPA buffer containing 0.1 M sodium fluoride (NaF, Sigma), 0.1 M β-glycerophosphate (Sigma), protease inhibitor cocktail (Sigma), and 1 mmol/L phenylmethylsulfonyl fluoride (PMSF, Sigma). Western blot analysis was performed as previously described^33^. Primary antibodies against ATM (sc-23921), Cdc2 (sc-8395), Keap1 (sc-365626), and β-actin (sc-47778) were obtained from Santa Cruz Biotechnology (TX. USA); antibodies against p-ATR (#2853), ATR (#13934), p-Cdc2 (Tyr-15,#4539), p21^WAF1/CIP1^ (#2947), p-Akt (#9271), and Akt (#9272) were from Cell Signal Technology (MA, USA); antibodies against Cyclin B1 (ab32053), p-ATM (S1981, ab81292), and p62/SQSTM1 (ab56416) were from Abcam; and antibodies against γH2AX (NB100-384), LC3B (NB100-2220), and ATG5 (NB110-53818) were purchased from Novus (CO, USA). Antibody for Nrf2 was purchased from Thermo Fisher (PA5-27882).

### Immunofluorescence staining

For immunofluorescence staining for γH2AX, cells were plated onto gelatin-coated glass coverslips in 6-well plates. Twenty-four or forty-eight hours after drug incubation, cells were fixed in 4% paraformaldehyde for 10 min and blocked with 3% BSA in 1X PBS. Cells were then incubated with anti-γH2AX antibody (NB100-384, Novus) for 1.5 h and examined after incubation with Alexa 568-conjugated anti-rabbit antibody for 1 h. Nuclear counterstaining was performed using 4′6-diamidino-2-phenylindole (DAPI, Sigma). Stained cells were examined under a confocal microscope (X400, LSM700, Carl Zeiss, Germany).

### Active caspase-3 assay

To assess apoptotic cell death, cells were treated with CQ and CDDP for 48 h and caspase-3 activity in the cell lysates was measured using a caspase-3 (cleaved) human ELISA kit (Invitrogen). ELISA was performed according to the manufacturer’s protocol.

### Cell cycle analysis by flow cytometry

Cells were plated at 3 × 10^5^ cells into 60 mm-dishes and treated with drugs for 24 and 48 h. Cells were trypsinized, washed with cold PBS, and fixed in 70% ethanol overnight at −20 ^o^C. Cells were washed with cold PBS and stained with propidium iodide (PI, Sigma) solution containing 50 µg/ml PI, 0.1 mg/ml RNase A (Sigma), and 0.05% Triton X-100 in PBS for 30 min at 37 ^o^C. Cells were washed once with cold PBS and cell cycle analysis was performed using FACSVerse^TM^ (BD FACS: Fluorescence-activated cell sorting, CA, USA).

### Transfection of plasmid expressing HA-p21^WAF1/CIP1^

Plasmid expressing HA-p21^WAF1/CIP1^ was purchased from Addgene. Plasmid encoding HA-p21^WAF1/CIP1^ or empty vector was transfected into cells (2 × 10^5^/well in 6-well plate) using Lipofectamine 2000 (Invitrogen). At 7 h after transfection, media was changed and drugs were treated to cells for an additional 36 h. After lysing cells in RIPA buffer, western blotting was performed.

### Hoechst/propidium iodide staining

A2780-CP20 cells (1 × 10^6^/dish) were plated in a 60 mm-dish and incubated with CDDP, CQ, or CQ combined with CDDP for 48 h. After incubation, culture media was collected in 15-ml falcon tubes and cells were washed with 1X cold PBS. Cells were trypsinized, were combined with culture media collected before trypsin treatment, and were washed twice with 1X cold PBS. Cells were incubated with Hoechst33342 and propidium iodide (Vybrant Apoptosis assay kit#5, ThermoFisher) for 10 min. Apoptosis and necrosis were measured with FACS Aria (BD Bioscience).

### Development of in vivo cell line xenograft and PDX models

In vivo animal study was reviewed and approved by the Institutional Animal Care and Use Committee at Samsung Biomedical Research Institute (protocol no. H-A9-003), which is permissioned by the Association for the Assessment and Accreditation of Laboratory Animal Care International and compliant with the guidelines of the Institute of Laboratory Animal Resources. Female BALB/c nude mice were obtained from Orient Bio (Seongnam, South Korea). Orthotopic models were prepared with A2780-CP20 cells (1 × 10^6^ cells/0.2 ml Hank’s balanced salt solution) by injection into the peritoneal cavities of BALB/c nude mice^34^. A PDX model of ovarian cancer was established using 6 to 8 weeks old mice as described in our previous report^35^. This study with PDX mouse model was approved by the Samsung Medical Center Institutional Review Board (file#201004004) and performed in accordance with approved guidelines and regulations. Orthotopic cell-line xenograft and PDX models were randomly divided into 4 groups (*n* = 10 per group) before administrating drugs. CDDP (4 mg/kg, once a week), CQ (50 mg/kg, three times a week), CQ and CDDP, or PBS were injected into model mice for the subsequent 3 weeks. Tumor development was monitored daily and mice were sacrificed at the end of treatment. Body weight and tumor weight were measured.

### Immunohistochemistry

For immunohistochemical staining of γH2AX as a marker for DNA damage, p21^WAF1/CIP1^, and Ki-67 as a marker for cell proliferation, tissue sections (4 µm-thick) were incubated with anti-γH2AX rabbit polyclonal antibody (NB100-384, Novus), anti-p21^WAF1/CIP1^ antibody (#2947, Cell signaling), or anti-Ki67 rabbit polyclonal antibody (NB600-1252, Novus) overnight at 4 °C in a humidity chamber after heat-induced epitope retrieval (HIER) with citrate buffer (pH 6.0; Dako, CA, USA). The sections were then incubated with HRP-conjugated secondary antibody against rabbit IgG (P-0448, Dako) for 30 min at RT. The color reaction was developed using the ready-to-use DAB (3,3’-diaminobenzidine) substrate-chromogen solution (Dako) for 5 min and sections were counterstained with Mayer’s hematoxylin (Sigma) for 30 s before dehydration and mounting. Slides were scanned with Scanscope microscopy (Aperio, Germany).

### Statistical analysis

Data were analyzed with Mann–Whitney *U* test and one-way analysis of variance (ANOVA) followed by the Newman–Keuls multiple comparison tests, as appropriate, using a statistical software package (Prism, GraphPad, CA, USA). *P* values less than 0.05 were considered statistically significant.

## Results

### CQ increases CDDP-induced cell death in EOC cells

**Fig. 1:**
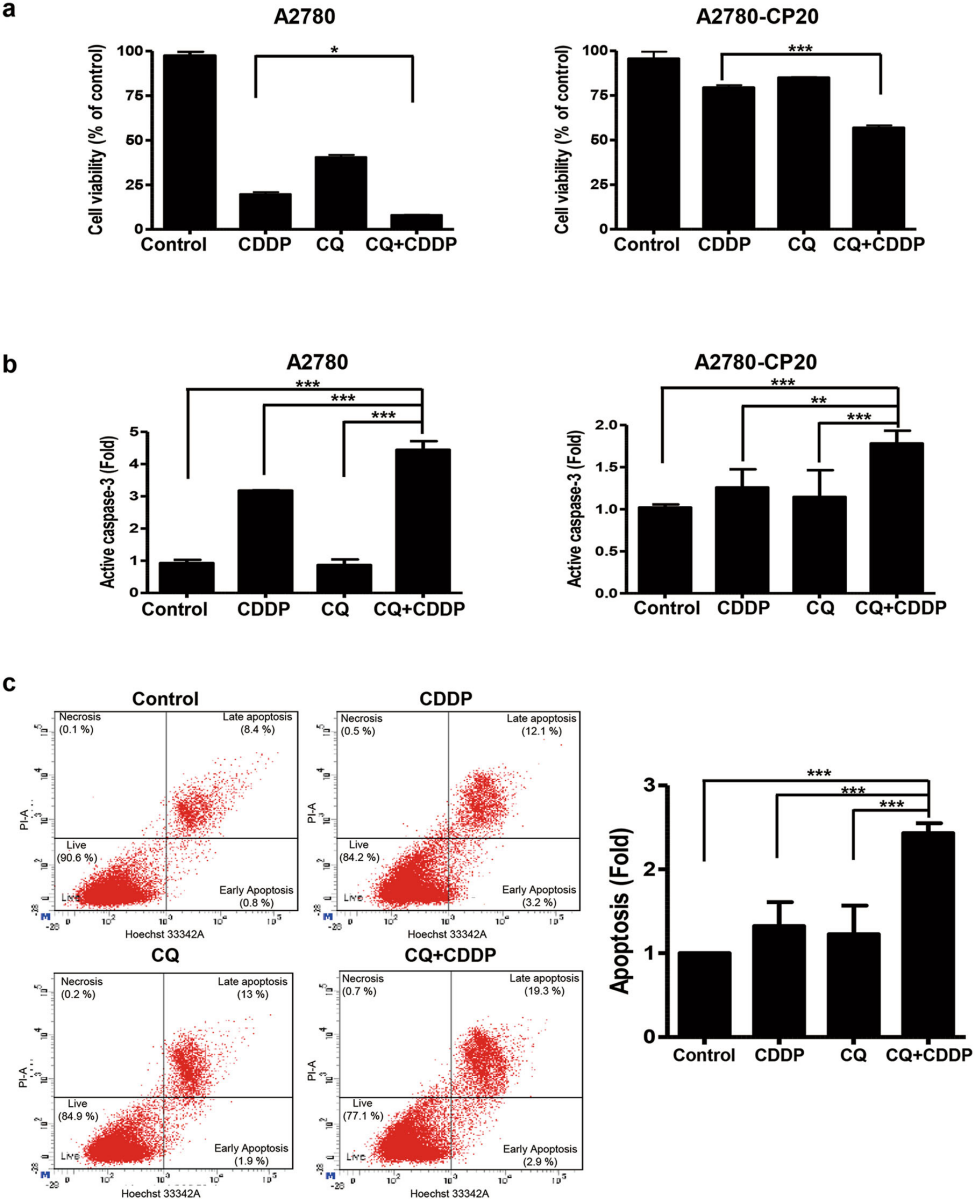
CQ sensitized EOC cells to CDDP. **a** CDDP-sensitive (A2780) and CDDP-resistant (A2780-CP20) EOC cells were treated with CDDP and CQ for 72 h, and cell viability was measured by MTT assay. Results are demonstrated by a bar graph. **b** Apoptotic cell death was measured by ELISA for detecting active caspase-3. A2780 and A2780-CP20 cells were treated with CDDP (1 µM and 5 µM, respectively) and CQ (20 µM and 30 µM, respectively) as indicated for 48 h, and cell lysates were used for caspase-3 assay. Results are shown as the mean ± SD of triplicate observations from three experiments (*n* = 3, *P < 0.05, **P < 0.01, ***P < 0.001). **c** A2780-CP20 cells were stained with Hoechst/propidium iodide to detect the contribution to apoptosis and necrotic cell death after treatment with CDDP and CQ for 48 h. The representative FACS data obtained from three experiments and a bar graph representing apoptotic cells are shown as the mean ± SD (*n* = 3, ***P < 0.001).

Lethal concentration 50 (LC50) of CQ or CDDP in the EOC cell lines A2780, A2780-CP20, and RMG-1 was investigated after 72 h treatment with each drug (Supplementary Fig. S1). In A2780 cells, 10 μM of CDDP induced cell death in more than 80% of cells, and the combination of CDDP with CQ further increased CDDP-induced cell death (Fig. 1a). However, in A2780-CP20 and RMG-1 cells, 10 μM of CDDP induced cell death in 20 and 46% of cells, respectively, and the CQ-CDDP combination induced cell death by 50 and 70% compared with controls (Fig. 1a and Supplementary Fig. S2a).

In apoptosis assays measuring active caspase-3, different amounts of CDDP were used for drug-sensitive A2780 and drug-resistant A2780-CP20 and RMG-1 cells (1 μM and 5 μM, respectively). CQ-CDDP combination significantly increased apoptosis in all cells (Fig. 1b and Supplementary Fig. S2b). Interestingly, in A2780-CP20 and RMG-1 cells, this combination more profoundly increased apoptosis compared with CQ or CDDP alone. To characterize the contribution of apoptosis/necrosis in this experimental condition, the apoptotic and necrotic cells were analyzed by Hoechst/propidium iodide staining (Fig. 1c). Although the proportion of necrosis was 0.1, 0.5, 0.2, and 0.7% of total cells in the control, CDDP, CQ, and CQ-CDDP treatments, respectively, apoptosis was increased in the CQ-CDDP combination compared with the control, consistent with the result of caspase-3 assay presented in Fig. 1b.

### CQ increases CDDP-induced DNA damage

**Fig. 2:**
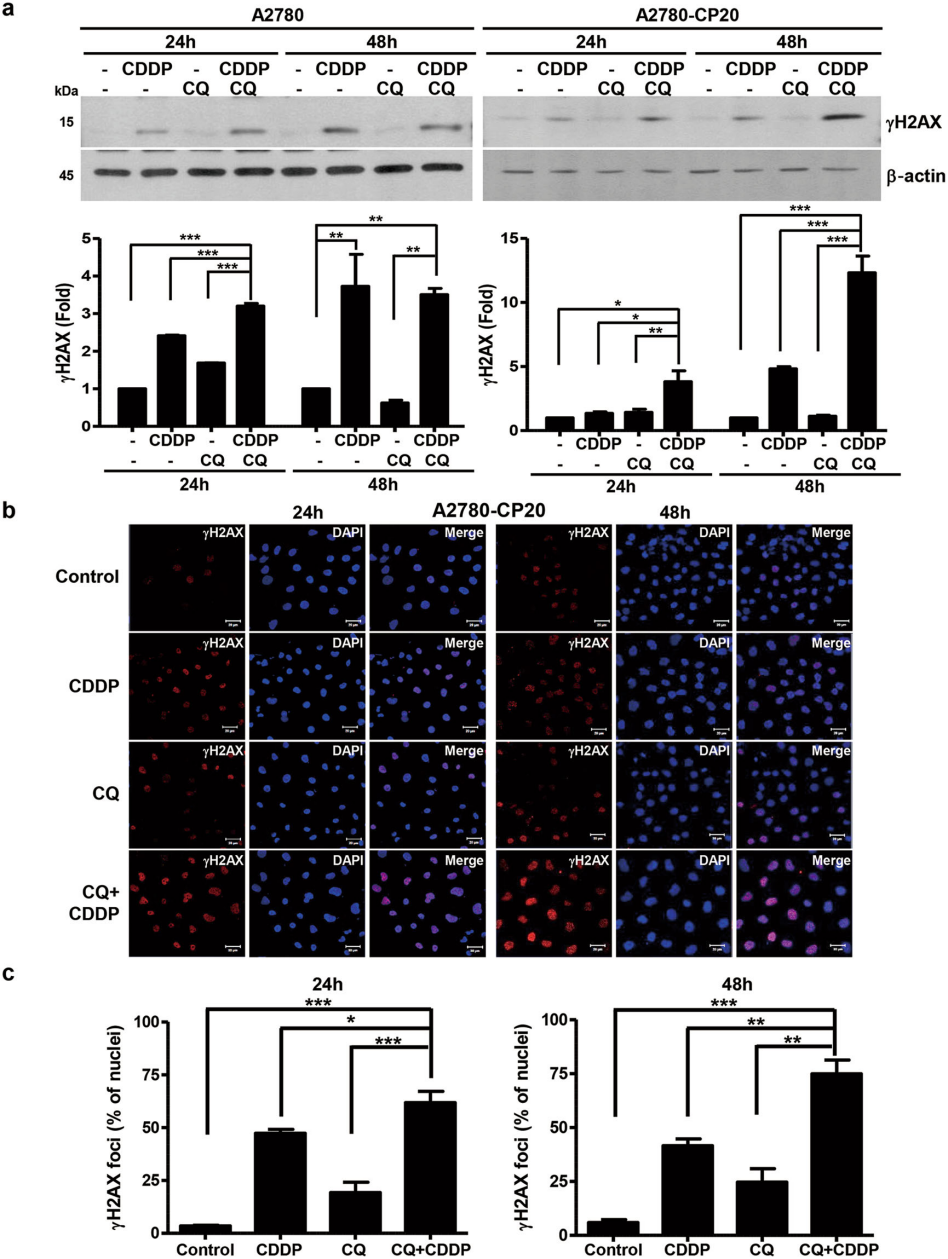
CQ increased CDDP-induced DNA damage in EOC cells. **a** EOC cells were treated with either CDDP, CQ, or the combination of CQ and CDDP for 24 h and 48 h. Phosphorylation of histone H2AX (γH2AX) was detected by western blot using anti-γH2AX antibody. **b** Nuclear foci formation of γH2AX was determined by immunofluorescent staining in A2780-CP20 cells treated with each drug as indicated (X400, scale bar represents 20 μm). DAPI was used for nuclear staining. **c** Nuclear foci formation of γH2AX obtained from **b** was quantified by a bar graph after calculating the number of nuclear foci of γH2AX and nuclei. The bar graph was generated with data obtained from three experiments (*n* = 3, *P < 0.05, **P < 0.01, ***P < 0.001).

CDDP induces DNA damage, leading to DNA damage-mediated cancer cell death^5^. The effect of the CQ-CDDP combination on DNA damage was studied by measuring amount of γH2AX. Treatment of CDDP and the combination with CQ induced γH2AX in A2780 cells in a time-dependent manner. However, in A2780-CP20 and RMG-1 cells, the combination with CQ increased γH2AX compared with CDDP alone at 48 h (Fig. 2a and Supplementary Fig. S2c). Induction of γH2AX by the CQ-CDDP combination was also determined by immunofluorescence staining in A2780-CP20 cells at 24 h and 48 h and demonstrated that the combination further increased γH2AX foci in nuclei, representing increased DNA damage (Fig. 2b, c).

### The CQ-CDDP combination arrests the cell cycle at G2/M and regulates DNA damage-related signaling proteins

**Fig. 3:**
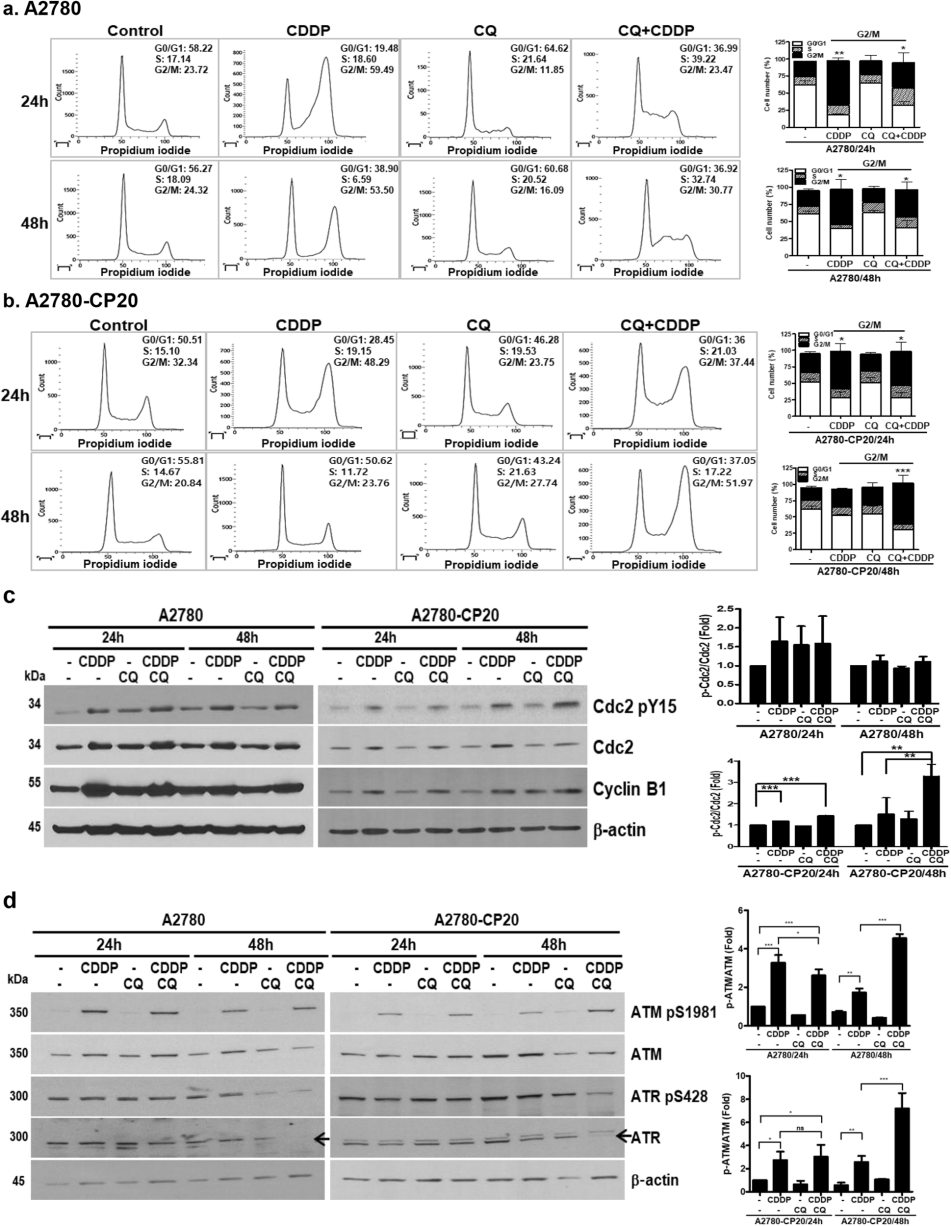
The CQ-CDDP combination arrested the cell cycle at G2/M and regulated ATM/ATR. **a** A2780 and **b** A2780-CP20 cells were incubated with each drug or the combination of drugs for 24 h and 48 h. Cells were stained with propidium iodide, and cell cycle distribution was measured by flow cytometry. Flow cytometry was performed three times, and representative data are presented. Cell cycle distribution was demonstrated by a bar graph and the statistical data represented for G2/M-arrested cell numbers compared with the control-treated cells. **c** Activation and expression of cell cycle-related proteins (Cdc2 and cyclin B1) were studied by western blot analysis in A2780 and A2780-CP20 cells using anti-phospho-specific antibody and antibodies recognizing the total proteins. The amount of phosphorylation of Cdc2 normalized by the amount of total Cdc2 expression in A2780 and A2780-CP20 cells is represented by a bar graph in the upper panel and lower panel, respectively. **d** DNA damage-recognizing proteins, ATM and ATR, were analyzed in A2780 and A2780-CP20 cells by western blot using phospho-specific antibodies and antibodies recognizing both the phosphorylated and unphosphorylated proteins. Arrow indicates ATR. Western blotting was performed at least three times, and a representative figure is presented. The amount of phosphorylation of ATM normalized by total ATM expression in A2780 and A2780-CP20 cells is represented by a bar graph in the upper panel and lower panel, respectively (*n* = 3, mean ± SD, *P < 0.05, **P < 0.01, ***P < 0.001).

The cell cycle is arrested upon DNA damage to allow the cell to repair the damage. Therefore, we assessed the cell cycle of EOC cells by FACS analysis after drug treatment. CDDP treatment for 24 h arrested the cell cycle of A2780 cells at G2/M, and this cell cycle arrest persisted until 48 h (Fig. 3a). The combination of CQ-CDDP in A2780 cells induced G2/M arrest with increasing incubation time. Interestingly, CQ-CDDP combination also induced S phase arrest at 24 h and slightly decreased it at 48 h. In A2780-CP20 cells, although 24 h treatment of CDDP also arrested the cell cycle at G2/M, this arrest was reversed during 48 h treatment (Fig. 3b). In contrast, the CQ-CDDP combination induced G2/M arrest in A2780-CP20 cells at 24 h, and the prolonged incubation further increased the number of G2/M-arrested cells (from 37% of G2/M-arrested cells at 24 h to 52% at 48 h). RMG-1 cells showed similar responses to A2780-CP20 cells (Supplementary Fig. S3a).

To determine the mechanism involved in the G2/M cell cycle arrest induced by the CQ-CDDP combination, we examined the phosphorylation of Cdc2 at tyrosine 15 (Cdc2-pY15, the inactive form), which is known to lead to G2/M arrest. CDDP induced Cdc2 expression in both A2780 and A2780-CP20 cells. Amount of Cdc2-pY15 was increased during 48 h treatment of the CQ-CDDP combination in all cells (Fig. 3c and Supplementary Fig. S3b). The ratio of p-Cdc2/Cdc2 was increased in CQ-CDDP-treated A2780-CP20 cells with 48 h incubation. However, in A2780 cells, treatments of CDDP and CQ-CDDP combination induced Cdc2 protein expression but did not significantly increase its phosphorylation. The error bars in A2780 cells show lack of statistical significance (Fig. 3c upper panel). Expression of Cyclin B1 was increased by CDDP and had no effect by CQ-CDDP combination in either A2780 or A2780-CP20 cells. We also evaluated upstream signaling of Cdc2. ATM, a DNA damage-recognition protein, was activated by CDDP in EOC cells. Although the CQ-CDDP combination inhibited ATM protein expression, the combination induced phosphorylation of ATM in all EOC cells compared with CDDP alone (Fig. 3d and Supplementary Fig. S3c). Although CDDP slightly induced phosphorylation of ATR, the CQ-CDDP combination had no effect on phosphorylation of ATR at 48 h in all EOC cells (Fig. 3d and Supplementary Fig. 3c). However, ATR protein expression was highly decreased by the CQ-CDDP combination. Taken together, these results show that the CQ-CDDP combination might induce G2/M cell cycle arrest through activation of ATM and inhibition of Cdc2.

### The CQ-CDDP combination has similar effects to ATM and ATR inhibitors

**Fig. 4:**
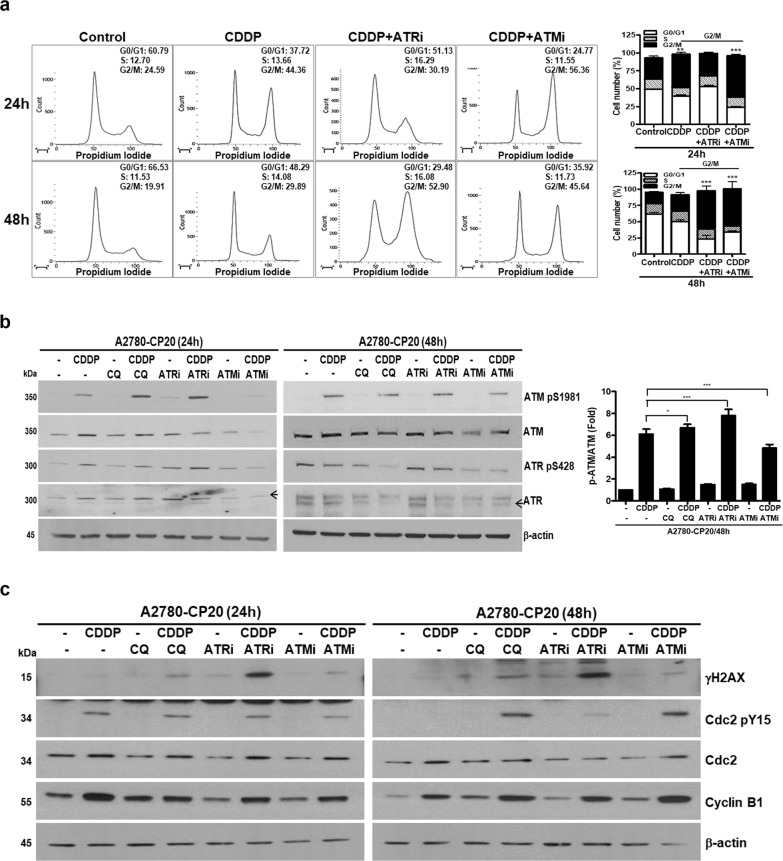
CQ had a similar effect to ATM or ATR inhibitor in combination with CDDP on the cell cycle. **a** Cell cycle distribution was analyzed by flow cytometry in A2780-CP20 cells treated with CDDP alone or in combination with ATM or ATR inhibitor for 24 h and 48 h. Flow cytometry was performed three times, and representative data are presented. Cell cycle distribution was demonstrated by a bar graph and the statistical data represented for G2/M-arrested cell numbers compared with the control cells. **b** A2780-CP20 cells were incubated with CDDP, CQ, ATR inhibitor, ATM inhibitor, or the combination of CDDP with either CQ, ATRi, or ATMi for 24 h and 48 h. Western blot for ATM and ATR was performed using antibodies for phospho-specific and antibodies recognizing both the phosphorylated and unphosphorylated proteins as indicated. The amount of phosphorylation of ATM normalized by the amount of total ATM expression is represented by a bar graph in the right panel. Arrow indicates ATR. **c** Expression of cell cycle-related proteins was determined in A2780-CP20 cells after 24 h and 48 h treatment with the CQ-CDDP combination. Western blot analysis was performed three times, and the representative data are presented (*n* = 3, mean ± SD, *P < 0.05, **P < 0.01, ***P < 0.001).

We compared the effects of ATM or ATR inhibitors with the CQ-CDDP combination on cell cycle and expression of signaling proteins. Inhibitors specific for ATM (ATMi) or ATR (ATRi) were incubated either alone or in combination with CDDP in A2780-CP20 cells. ATMi alone seemed to induce G2/M arrest by about 1.5-fold compared with the control at 48 h incubation but this induction was not statistically significant as calculated from three experiments (Supplementary Fig. S4). However, in combination with CDDP, these inhibitors significantly induced G2/M arrest (Fig. 4a). The pattern of G2/M arrest by the CQ-CDDP combination in A2780-CP20 cells was similar to that of the combination of ATMi or ATRi with CDDP, in that G2/M arrest lasted until 48 h. ATMi inhibited CDDP-induced ATM phosphorylation at 48 h, demonstrating that the inhibitor successfully inhibited its target protein (Fig. 4b). Combination of ATRi with CDDP highly increased γH2AX expression compared with CDDP or inhibitor alone. Combination of ATMi with CDDP increased Cdc2-pY15 at 48 h incubation, which was the same effect as with the CQ-CDDP combination (Fig. 4c).

### CQ increases CDDP-induced cell death via inhibition of autophagy and induced p21^WAF1/CIP1^ protein expression

**Fig. 5:**
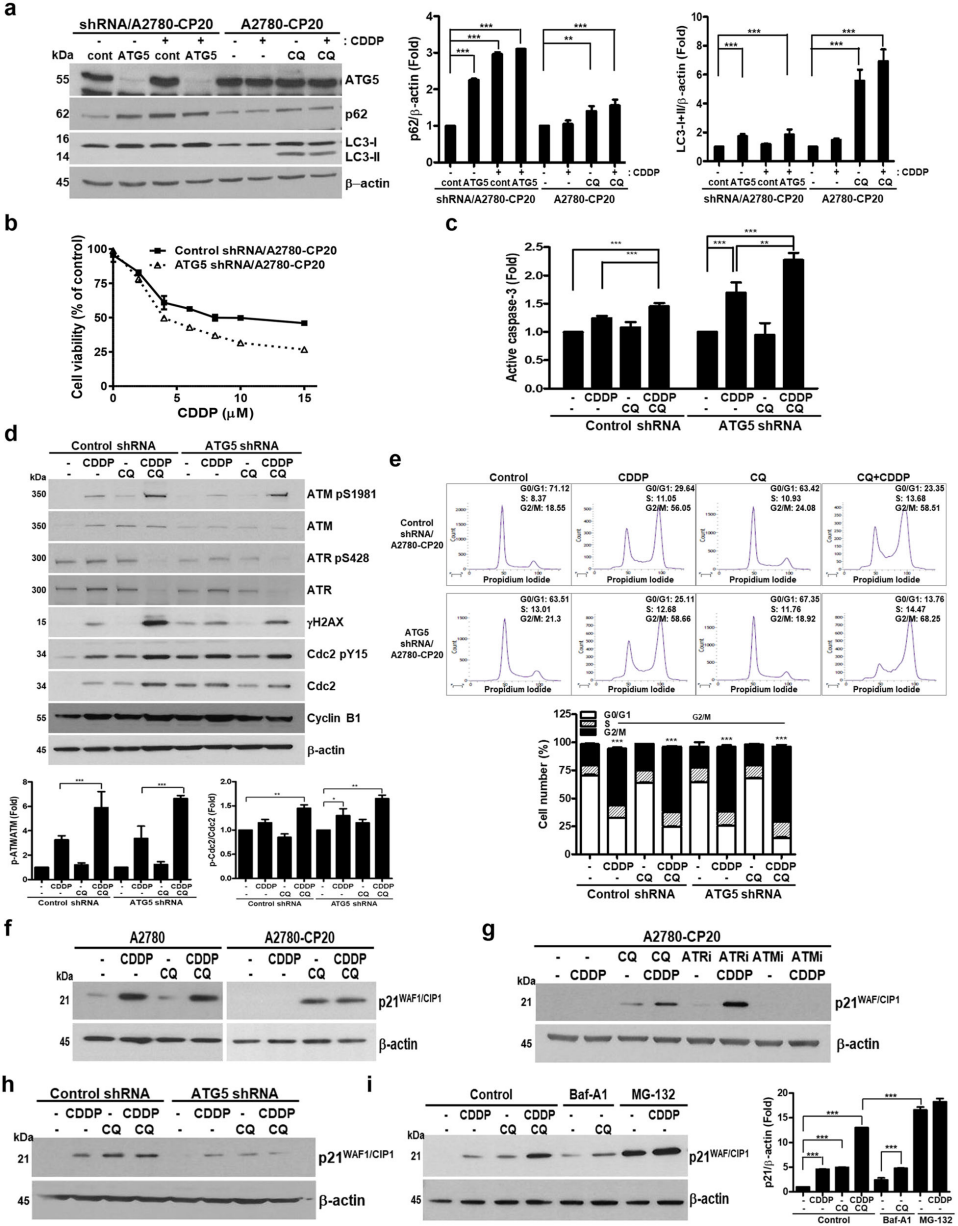
CQ increased CDDP-induced cell death in drug-resistant EOC through autophagy inhibition and induction of p21^WAF1/CIP1^. **a** A2780-CP20 cells stably expressing control or ATG5 shRNA were treated with CDDP for 48 h, and the expression of ATG5 and autophagy marker proteins, LC3 and p62, were analyzed by western blot using specific antibodies. Knockdown efficiency of ATG5 was shown by western blot. Expression of LC3 and p62 was also analyzed by western blot in A2780-CP20 cells treated with CDDP, CQ, or the CQ-CDDP combination for 48 h. β-actin was used for a protein-loading control. shRNA/CP20 represents control-shRNA- or ATG5-shRNA-expressing A2780-CP20 cells. Western blot was performed three times, and representative data are presented (left panel). The amounts of p62 and LC3-I and -II normalized to the amount of β-actin are represented by a bar graph (middle and right panels, respectively). **b** Cell viability was measured by MTT assay in A2780-CP20 cells stably expressing control- (closed squares) or ATG5-shRNA (open triangles) in the presence of different concentrations of CDDP at 72 h. **c** Apoptotic cell death was measured by ELISA for detecting active caspase-3. Control-shRNA and ATG5-shRNA/A2780-CP20 cells were treated with CDDP and CQ as indicated for 48 h, and cell lysates were used for caspase-3 assay. Results are shown as the mean ± SD of duplicate observations from three experiments (*n* = 3, **P < 0.01, ***P < 0.001). **d** To analyze DNA damage recognition proteins and downstream cell cycle-related proteins, cell lysates were analyzed on a 4–12% gradient SDS-PAGE by western blot in shRNA/A2780-CP20 stable cells after incubation with CDDP, CQ, and CQ-CDDP combination for 48 h. Amounts of phosphorylated ATM and Cdc2 normalized by the amount of each total protein are represented by a bar graph in the lower panels. **e** Cell cycle distribution of A2780-CP20 stable cells expressing either control- or ATG5-shRNA was analyzed after incubation with CDDP, CQ or CQ combined with CDDP for 48 h (upper panel). Flow cytometry was performed for three experiments, and representative data are shown. Cell cycle distribution was represented by bar graphs and the statistical data demonstrated cell number in G2/M (lower panel) compared with the control. **f** Expression of p21^WAF1/CIP1^ was analyzed by western blot of A2780 and A2780-CP20 cells after incubation with CDDP, CQ, or CQ-CDDP combination. **g** A2780-CP20 cells were incubated with CDDP, CQ, ATR inhibitor, ATM inhibitor, or the combination of CDDP with either CQ, ATRi, or ATMi for 48 h. Expression of p21^WAF1/CIP1^ was analyzed by western blot. **h** Expression of p21^WAF1/CIP1^ was determined by western blot in shRNA/A2780-CP20 stable cells after incubation with CQ for 48 h. Expression of p21^WAF1/CIP1^ was determined by western blot analysis in control shRNA and ATG5 shRNA/A2780-CP20 cells treated with drugs as indicated. **i** A2780-CP20 cells were incubated with CDDP, CQ, bafilomycin A1 (Baf-A1), CQ-Baf-A1 combination, MG-132, or the combination of CDDP with CQ or MG-132 as indicated. CQ and CDDP were treated for 48 h and Baf-A1 and MG-132 were treated overnight. Western blot was performed using anti-p21^WAF1/CIP1^ antibody. Western blot analysis was performed at least three times, and representative data are presented in this study (mean ± SD, *P < 0.05, **P < 0.01, ***P < 0.001).

We next evaluated whether the inhibition of autophagy by CQ is involved in CDDP-induced cell death. To inhibit autophagy, lentivirus expressing ATG5-specific shRNA was generated and used to infect A2780-CP20 cells (ATG5-shRNA cells). Knockdown of ATG5 was analyzed by western blot, and expression of p62 was increased in ATG5-shRNA cells and CQ-treated A2780-CP20 cells, indicating autophagy inhibition (Fig. 5a). Conversion from LC3-I to LC3-II was inhibited in ATG5-shRNA cells compared with the control cells determined by increased amount of LC3-I. In contrast, since CQ inhibits fusion between autophagosome and lysosome^16^, LC3-II accumulated in cells treated with CQ. Cell viability of ATG5-shRNA or control-shRNA cells were measured by MTT assay after incubation with different amounts of CDDP. In contrast with control cells, CDDP at 4 µM resulted in 50% cell death in ATG5-shRNA cells, demonstrating that inhibition of autophagy sensitized A2780-CP20 cells to CDDP (Fig. 5b). In caspase-3 assay, CDDP in ATG5-shRNA cells induced caspase-3 activity compared with CDDP-treated control-shRNA cells, and this cell death increase was further induced by CQ-CDDP treatment in ATG5-deficient cells (Fig. 5c). Interestingly, CQ alone had no significant effect on caspase-3 activity.

To study the effect of autophagy inhibition on DNA damage/cell cycle-related proteins, western blot analysis was performed. Total expression of ATM was inhibited by knockdown of ATG5. Treatment with CDDP in ATG5-shRNA cells induced phosphorylation of ATM compared with control-treated ATG5-shRNA cells (Fig. 5d). CQ-CDDP combination further induced phosphorylation of ATM. Although ATR expression was inhibited by CQ-CDDP combination in both control- and ATG5-shRNA cells, its phosphorylation was not affected. Knockdown of ATG5 induced expression of γH2AX, demonstrating that inhibition of autophagy enhanced DNA damage. Treatment of CDDP further induced γH2AX in ATG5-shRNA and control-shRNA cells. Interestingly, treatment of CQ-CDDP combination in ATG5-shRNA cells induced γH2AX to a lesser extent than the control-shRNA cells treated with CQ-CDDP combination. CQ alone had no effect on γH2AX expression. Cdc2-pY15 was increased by CQ-CDDP combination in ATG5-shRNA cells, demonstrating inhibition of Cdc2 by inhibition of autophagy. Cdc2 and cyclin B1 are involved in G2/M cell cycle progression, and the effect of inhibition of autophagy on cell cycle distribution was assessed. G2/M arrest was increased in ATG5-shRNA cells treated with CDDP and was further induced by CQ-CDDP combination (Fig. 5e).

p21^WAF1/CIP1^ is activated by DNA damage and induced G2/M arrest^23^; therefore, the effect of CQ-CDDP on p21^WAF1/CIP1^ expression was investigated. In A2780 and RMG-1 cells, protein expression of p21^WAF1/CIP1^ was highly induced by CDDP (Fig. 5f and Supplementary Fig. S5, respectively). However, p21^WAF1/CIP1^ expression was highly induced by CQ alone and the CQ-CDDP combination in A2780-CP20 cells. We examined whether ATMi or ATRi inhibitors could induce p21^WAF1/CIP1^. Induction of p21^WAF1/CIP1^ was hardly observed in cells treated with ATRi alone but ATRi combined with CDDP highly induced its expression (Fig. 5g). However, ATMi alone and ATMi-CDDP combination had no effect on induction of p21^WAF1/CIP1^. To determine whether autophagy inhibition induced p21^WAF1/CIP1^, expression of p21^WAF1/CIP1^ was assessed in ATG5-shRNA cells (Fig. 5h). In control-shRNA cells, single treatment of CDDP and CQ induced p21^WAF1/CIP1^, and its expression was further enhanced upon treatment with CQ-CDDP combination. However, although knockdown of ATG5 induced p21^WAF1/CIP1^ by CDDP, CQ, and CQ-CDDP combination, it was not as strong as in the control-shRNA cells, demonstrating that p21^WAF1/CIP1^ was not efficiently induced in ATG5-shRNA cells. Another autophagy inhibitor, Bafilomycin A1 (Baf-A1), was used to examine effect of autophagy on induction of p21^WAF1/CIP1^. Induction of p21^WAF1/CIP1^ was compared with that in cells treated with CQ-CDDP, Baf-A1, the combination of Baf-A1-CQ, the proteasome inhibitor MG-132, or co-treatment with MG-132 and CDDP (Fig. 5i). Baf-A1 alone slightly induced p21^WAF1/CIP1^ expression, while Baf-A1-CQ combination further induced its expression. However, treatment of MG-132 or its combination with CDDP strongly induced p21^WAF1/CIP1^. These findings suggest that enhanced p21^WAF1/CIP1^ expression by the CQ-CDDP combination may be due to inhibition of both autophagy and the proteasome by CQ.

### CQ-CDDP-induced cell death was partially dependent on induction of p21^WAF1/CIP1^

**Fig. 6:**
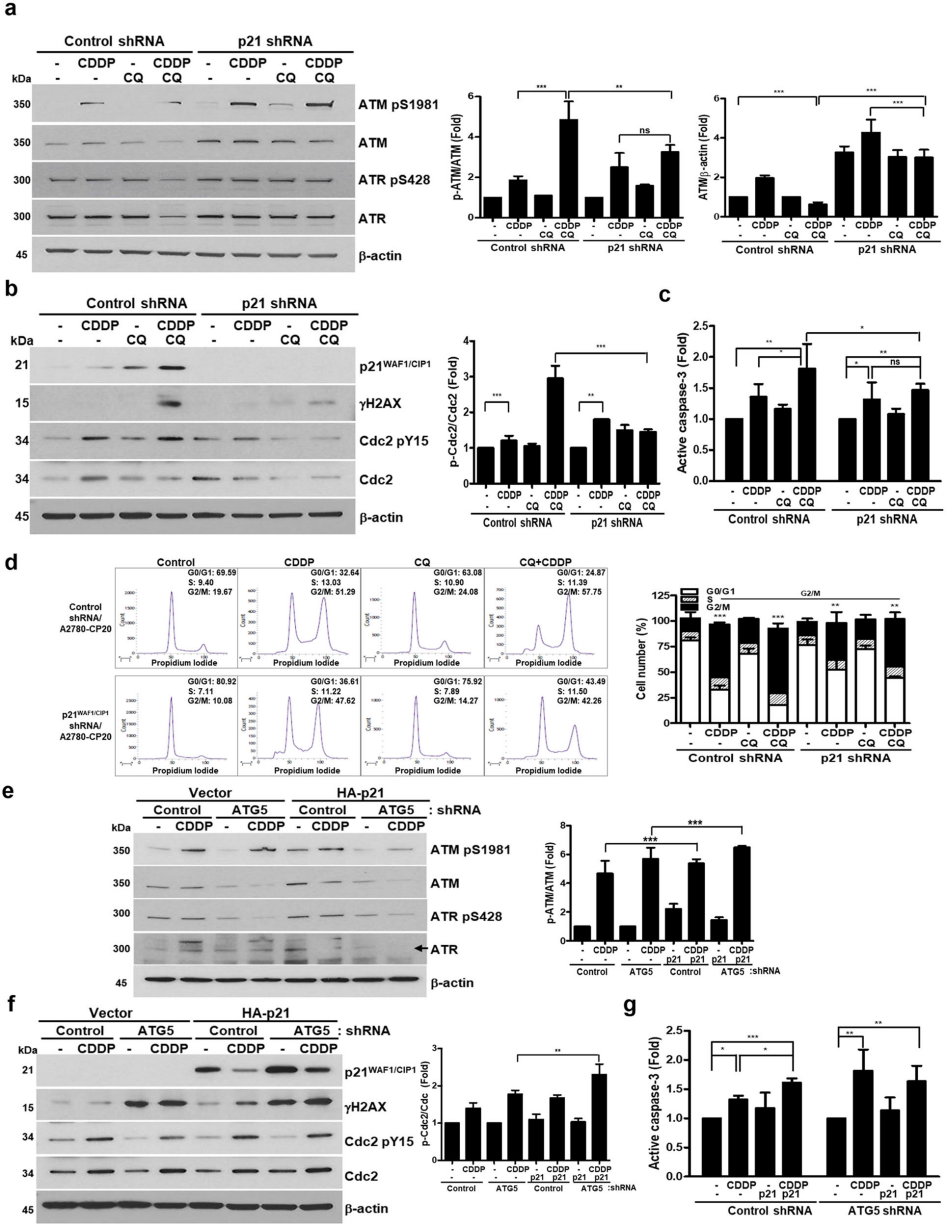
Expression of p21^WAF1/CIP1^ was important for the cytotoxic effect of CQ-CDDP. **a, b** Proteins related to DNA damage and cell cycle progression were analyzed by western blot in control-shRNA and p21^WAF1/CIP1^-shRNA/A2780-CP20 cells. The amount of ATM normalized by β-actin is represented by a bar graph. Amounts of phosphorylated ATM and Cdc2 normalized by the amount of each total protein are represented by a bar graph in the right panel (upper and lower panels, respectively). **c** Active caspase-3 was measured in control-shRNA and p21^WAF1/CIP1^-shRNA cells after treatment of drugs for 48 h as indicated. Results are shown as the mean ± SD of triplicate observations from three experiments. **d** Cell cycle distribution of A2780-CP20 stable cells expressing either control- or p21^WAF1/CIP1^-shRNA was analyzed after incubation with CDDP, CQ, or CQ combined with CDDP for 48 h. Flow cytometry was performed for three experiments, and representative data are shown. Cell cycle distribution was represented by bar graphs and the statistical data demonstrated cell number in G2/M (right panel) compared with the control. **e, f** HA-tagged p21^WAF1/CIP1^ or the empty vector were transfected into control-shRNA and ATG5-shRNA/A2780-CP20 cells. After transfection, cells were incubated with CDDP for 48 h. Expression of HA-tagged p21^WAF1/CIP1^ was analyzed by western blot using anti-p21^WAF1/CIP1^ antibody. Expression of proteins involved in DNA damage and cell cycle progression as well as γH2AX was detected by western blot using their specific antibodies. β-actin served as a protein-loading control. Arrow indicates ATR expression. **g** Caspase-3 activity was measured by detecting active caspase-3 in cells transfected with HA-tagged p21^WAF1/CIP1^ plasmid or the control vector, followed by incubation with CDDP. Results are shown as the mean ± SD of duplicate observations from three experiments (*n* = 3, mean ± SD, *P < 0.05, **P < 0.01, ***P < 0.001, ns not significant).

The functional significance of CQ-induced p21^WAF1/CIP1^ was studied by knockdown of p21^WAF1/CIP1^ with its specific shRNA in A2780-CP20 cells and by its ectopic expression with HA-tagged p21^WAF1/CIP1^. Knockdown efficiency of p21^WAF1/CIP1^ was analyzed by western blot (Fig. 6a). Proteins involved in DNA damage and cell cycle arrest were assessed in p21^WAF1/CIP1^-shRNA cells compared with control-shRNA cells. Combination of CQ-CDDP as well as CDDP and CQ single treatment induced ATM protein expression in p21^WAF1/CIP1^-shRNA cells compared with control cells. However, phosphorylated ATM was reduced in p21^WAF1/CIP1^-shRNA cells. ATR protein expression was also increased in CQ-CDDP-treated p21^WAF1/CIP1^-shRNA cells compared with CQ-CDDP-treated control cells. However, phosphorylation of ATR was not affected in p21^WAF1/CIP1^-shRNA cells. Expression of γH2AX was highly reduced by CQ-CDDP combination in p21^WAF1/CIP1-^shRNA cells (Fig. 6b). Cdc2-pY15 was also decreased in p21^WAF1/CIP1^-shRNA cells treated with CQ-CDDP compared with CQ-CDDP-treated control cells, demonstrating reduced inhibition of Cdc2 by knockdown of p21^WAF1/CIP1^. Caspase-3 activity in CQ-CDDP combination-treated cells was inhibited by about 20% by knockdown of p21^WAF1/CIP1^ compared with that of CQ-CDDP-treated control cells (Fig. 6c). Although knockdown of p21^WAF1/CIP1^ also induced G2/M arrest by CDDP and CQ-CDDP combination, the number of G2/M-arrested cells were reduced from 51.29% to 47.62% and from 57.75% to 42.26% in CDDP- and CQ-CDDP-treated p21^WAF1/CIP1^-shRNA cells compared with the control cells, respectively (Fig. 6d). Ectopic expression of HA-p21^WAF1/CIP1^ induced phosphorylation of ATM but decreased ATM expression in CDDP-treated ATG5-shRNA cells compared with CDDP-treated vector-transfected cells (Fig. 6e). In control-shRNA cells, HA-p21^WAF1/CIP1^ in the presence of CDDP induced γH2AX compared with CDDP-treated vector-transfected control-shRNA cells (Fig. 6f). In ATG5-shRNA cells, γH2AX was increased compared with control-shRNA cells and the enhanced expression was further increased by HA-p21^WAF1/CIP1^ and CDDP treatment. Ratio of p-Cdc2/Cdc2 was increased in CDDP-treated HA-p21^WAF1/CIP1^-transfected cells, demonstrating inhibition of Cdc2 by HA-p21^WAF1/CIP1^. Caspase-3 activity was increased in CDDP-treated HA-p21^WAF1/CIP1^-transfected cells compared with the CDDP-treated vector-transfected cells (Fig. 6g). These results demonstrate that overexpressed p21^WAF1/CIP1^ may partially replace the function of CQ in the presence of CDDP.

### The CQ-CDDP combination significantly decreases tumor weight in orthotopic and drug-resistant PDX mouse models for EOC

**Fig. 7:**
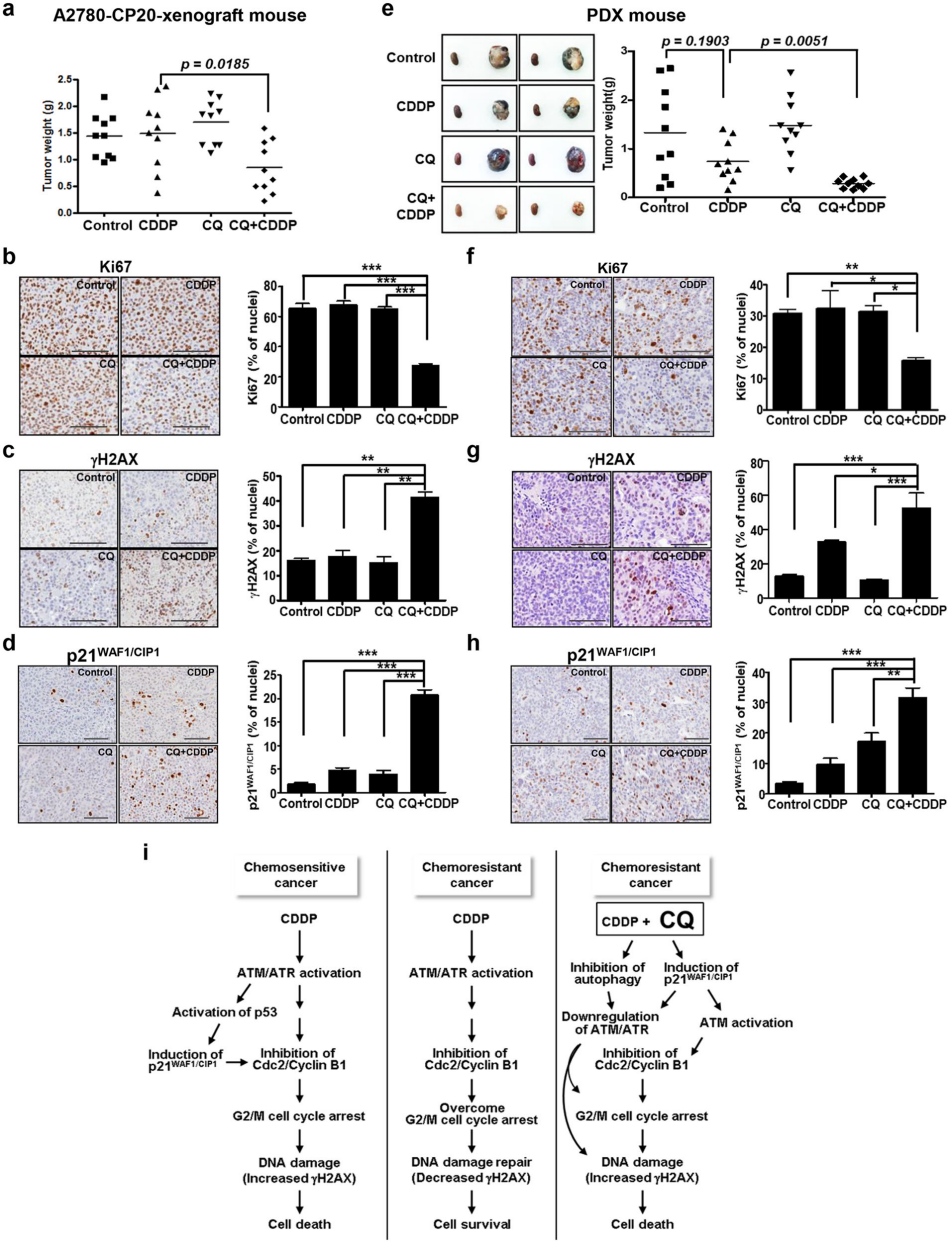
The CQ-CDDP combination significantly decreased tumor growth in drug-resistant EOC orthotopic xenograft and PDX models. **a** Drug-resistant EOC orthotopic mice were generated by i.p. injection of A2780-CP20 cells, and each drug was injected into the mice. After mice were sacrificed, tumors were isolated and weighed. Tumor weights are shown on a scatter plot. **b**–**d** Tumor tissues obtained from orthotopic mice were stained with antibodies against Ki67, a proliferation marker; γH2AX, a DNA damage marker protein; or p21^WAF1/CIP1^ (X200, left panels). Bar graphs (right panels) show numbers of Ki67-, γH2AX-, or p21^WAF1/CIP1^-stained nuclei relative to total hematoxylin-stained nuclei. Scale bar represents 200 μm. **e** EOC PDX mice were generated using subrenal implantation with a tumor from a patient with drug-resistant serous papillary adenocarcinoma; each drug or the combination of CQ and CDDP was injected into the peritoneal cavity of xenograft mice. Left panel shows tumor size compared with a kidney obtained from the same mouse, and right panel shows tumor weight on a scatter plot. **f**–**h** Tumor tissues obtained from PDX mice were stained with antibodies against Ki67, γH2AX, and p21^WAF1/CIP1^ (X200, left panels). Scale bar represents 200 μm. Bar graphs (right panels) show numbers of Ki67-, γH2AX-, or p21^WAF1/CIP1^-stained nuclei relative to total hematoxylin-stained nuclei. All data represent the mean ± SD (*P < 0.05, **P < 0.01, ***P < 0.001). **i** A proposed functional mechanism of CQ-CDDP in chemo-resistant EOC.

To test the efficacy of CQ-CDDP combination in vivo, we used an orthotopic mouse model established with A2780-CP20 cells (Fig. 7a–d) and a PDX model established with human EOC tissue^35^ (Fig. 7e–h). The human EOC tissue used in generation of a PDX model was from a 62-year-old woman with platinum resistance who was heavily pretreated with chemotherapy of paclitaxel-carboplatin, docetaxel, belotecan, and etoposide-ifosphamide and initially presented with FIGO stage IIIC, high-grade serous histology. The tumors obtained from orthotopic and PDX xenograft mice injected with the CQ-CDDP combination, were significantly smaller than those obtained from control, CDDP alone, and CQ alone groups (Fig. 7a and e, respectively). Immunohistochemical staining for Ki67, a proliferation marker protein, revealed that the numbers of proliferating cells in tumors obtained from the CQ-CDDP combination groups in orthotopic and PDX xenograft mice were reduced by 50 and 60% of that in controls, respectively (Fig. 7b and f). The combination group showed significantly increased immunostaining of γH2AX compared with the control groups (Fig. 7c and g). Expression of p21^WAF1/CIP1^ was increased in tumors from the CQ-CDDP combination group in orthotopic and PDX xenograft mice compared with the control groups (Fig. 7d and h, respectively).

## Discussion

In this study, we found that CQ increases cytotoxicity in combination with CDDP by inducing lethal DNA damage through induction of p21^WAF1/CIP1^ expression and autophagy inhibition in CDDP-resistant EOC.

A proposed functional mechanism of CQ-CDDP combination in drug-resistant EOC is presented in Fig. 7i. CDDP induces DNA damage and activates a signaling cascade involved in DNA damage/cell cycle regulation. In chemo-sensitive cancer, CDDP activates ATM/ATR signaling cascades by triggering DNA damage, activates p53 to induce p21^WAF1/CIP1^, arrests cell cycle, inhibits DNA repair, and finally leads to cell death. However, in chemo-resistant cancer, cells can survive CDDP-induced DNA damage by overcoming cell cycle arrest. Treatment with CQ combined with CDDP in chemo-resistant cancer induces p21^WAF1/CIP1^. Although total ATM expression is decreased, the induced p21^WAF1/CIP1^ activates ATM and inhibits Cdc2, leading to G2/M arrest. Expression of p21^WAF1/CIP1^ highly induces γH2AX, demonstrating increased DNA damage and potential cell death. Autophagy inhibitory function of CQ inhibits ATM/ATR expression but slightly induces activation of ATM and inhibition of Cdc2 and thereby induces persistent G2/M arrest. Increased DNA damage (detected by γH2AX expression level) by CQ-CDDP finally leads to cell death. Therefore, induction of p21^WAF1/CIP1^ and inhibition of autophagy may additively function in cytotoxicity of CQ-CDDP combination in drug-resistant EOC.

We also studied the effect of CQ-CDDP combination on signaling pathways related to autophagy and apoptosis. Akt regulates autophagy and apoptosis, and the effect of CQ-CDDP on Akt was determined (Supplementary Fig. S6). CDDP-induced Akt expression and activated Akt activity in A2780-CP20 cells as measured by phosphorylation of Akt using its phosphor-specific antibody. However, CQ inhibited CDDP-induced Akt activity and its expression. These results demonstrate that inhibition of Akt by CQ-CDDP combination may play a role in cytotoxicity of EOC.

The cytotoxic effect of CQ is related to mitochondrial dysfunction induced by increasing mitochondrial reactive oxygen species (ROS)^36^. Therefore, mitochondrial ROS was measured in A2780-CP20 cells treated with drugs using MitoSox kit (Supplementary Fig. S6b). CQ-induced mitochondrial ROS and this induction was further increased by CQ-CDDP combination. Nrf2 is an antioxidant protein, and its interacting protein, Keap1, is a negative regulatory protein of Nrf2 via induction of its degradation^37^. p62 (an autophagy marker protein) interacts with Keap1 and thereby inhibits degradation of Nrf2. Expression of Nrf2 and Keap1 was assessed (Supplementary Fig. S6c). CDDP induced Keap1 and CQ inhibited CDDP-induced Keap1 expression. Expression of Nrf2 was not significantly changed. These results suggest that CQ-induced mitochondrial ROS was not related to the expression level of Nrf2. Taken together, CQ-induced mitochondrial ROS may contribute to the cytotoxic effect of CQ when combined with CDDP.

Cellular senescence leads to permanent cell cycle arrest^38^. The effect of CQ on senescence was assessed by staining A2780 and A2780-CP20 cells with senescence-associated β-galactosidase after treatment with drugs as indicated (Supplementary Fig. S6d). In A2780 cells, CDDP induced cellular senescence, while CQ inhibited CDDP-induced senescence. In A2780-CP20 cells, CDDP did not significantly induce senescence. Thus, senescence may not be a critical factor for CQ-CDDP-mediated cytotoxicity in drug-resistant EOC.

Studies on inhibitors to target DNA damage/cell cycle checkpoint for therapeutic purposes in different type of cancers are ongoing^39–41^. Recently, inhibitors for ATM and ATR have been investigated in preclinical applications^42,43^. In this study, ATMi-CDDP combination increased inactive Cdc2 but did not induce γH2AX expression. H2AX is phosphorylated by ATM, ATR, and DNA-PK. CDDP activates ATM upon DNA damage^44^. Therefore, in the CDDP-induced DNA damage/repair signaling pathway, ATM is more likely to be important protein for γH2AX than other kinases. In contrast, ATRi-CDDP combination induced expression of γH2AX but inactivation of Cdc2 was not observed. The phosphorylated Cdc2 at Y15 is dephosphorylated by Cdc25C phosphatase and thereby is degraded. Cdc25C is inhibited by the ATR/Chk1 signaling pathway, and inhibition of ATR by its inhibitor may result in active Cdc25C and thereby enhanced Cdc2 degradation^45^. These results demonstrated that, although CQ, ATRi, and ATMi presented similar results in DNA damage/cell cycle signaling pathway in the presence of CDDP, the detailed functional mechanisms may differ in these drugs.

In this study, CQ and CQ-CDDP combination induced p21^WAF1/CIP1^ expression in drug-resistant EOC, and this induction was not sufficient in ATG5-shRNA cells compared with control-shRNA cells. It seems that p21^WAF1/CIP1^ may be degraded via a proteasome degradation pathway in drug-resistant EOCs since p21^WAF1/CIP1^ was highly increased in the presence of MG-132. In support of our data, proteasomal degradation of p21^WAF1/CIP1^ has been reported in several cell lines^46–48^. Furthermore, inhibition of lysosomal degradation by CQ led to inhibition of proteasomal degradation, but knockdown of ATG5 could not inhibit proteasomal activity^49,50^, consistent with our result that induction of p21^WAF1/CIP1^ was not sufficient in ATG5-shRNA cells. These study findings indicate that CQ may enhance p21^WAF1/CIP1^ expression by inhibition of proteasomal degradation and by partly its autophagy inhibition.

In conclusion, we found that CQ in combination with CDDP increased the cytotoxicity of drug-resistant EOC cells mainly by inducing lethal DNA damage through induction of p21^WAF1/CIP1^ expression and autophagy inhibition. This study suggests that CQ may be a promising drug for combination therapy with anti-cancer drugs to treat chemo-resistant EOCs.

## Supplementary information

Supplementary Materials and Methods

Supplementary Figure Legend

Supplementary Figure S1

Supplementary Figure S2

Supplementary Figure S3

Supplementary Figure S4

Supplementary Figure S5

Supplementary Figure S6
