# Supplementary material for: Chloroquine reverses chemoresistance via upregulation of p21WAF1/CIP1 and autophagy inhibition in ovarian cancer
Source: Cell Death Dis. 2020 Dec 4;11(12):1034. doi: 10.1038/s41419-020-03242-x (PMC7718923; doi:10.1038/s41419-020-03242-x)
Supplement: Supplementary file 1 — Supplementary Materials and Methods [file 41419_2020_3242_MOESM1_ESM.docx]

**Supplementary Materials and Methods**

**MTT assay**

For assaying cell viability, cells were plated at 3000~4000 cells/well in 96-well plates in triplicate. Cells were treated with CDDP, CQ, or the CQ-CDDP combination for 24, 48, or 72 h at 37^o^C in a 5% CO_2_ incubator. After treatment, cells were incubated with MTT solution (5 mg/ml in PBS, Sigma) for 4 h in a 37^o^C incubator. The MTT crystal was dissolved in DMSO (Duchefa Biochemie, Netherlands) and cell viability was measured at 540 nm by spectrometry. The data are presented as the mean±SEM from three different assays.

**Detection of Mitochondrial reactive oxygen species (ROS)**

A2780-CP20 cells were plated in a 6-well plate and incubated with CDDP, CQ, or CQ combined with CDDP for 48 h. After incubation, cells were washed twice with HBSS containing calcium and magnesium and stained with 5 μM MitoSox (Molecular Probes) for 40 min in CO_2_-incubator at 37^o^C protected from light. Cells were washed with HBSS and were stained with DAPI for 10 min. Stained cells were immediately observed under microscope.

**Senescence assay**

A2780 and A2780-CP20 cells were plated in a 6-well plate and were treated with CDDP, CQ, or CQ combined with CDDP for 48 h. Cells were washed with PBS and were stained with senescence-associated β-galactosidase cell staining kit (Cell signaling) according to the manufacturer’s instructions. Cells were incubated with staining solution at ~pH6.0 for 24 h at 37 ^o^C-dry incubator. Cells stained with β-galactosidase were observed under microscope.
