# Supplementary material for: Chloroquine reverses chemoresistance via upregulation of p21WAF1/CIP1 and autophagy inhibition in ovarian cancer
Source: Cell Death Dis. 2020 Dec 4;11(12):1034. doi: 10.1038/s41419-020-03242-x (PMC7718923; doi:10.1038/s41419-020-03242-x)
Supplement: Supplementary file 2 — Supplementary Figure Legend [file 41419_2020_3242_MOESM2_ESM.docx]

**Supplementary Figure Legends**

**Supplementary Fig. S1. Sensitivity to CQ and CDDP differed among the EOC cell lines.** **a** CDDP-sensitive (A2780) and CDDP-resistant (A2780-CP20 and RMG-1) EOC cells were treated with increasing concentrations of CQ for 72 h and cell viability was measured by MTT assay. **b** EOC cells were treated with increasing concentrations of CDDP for 72 h and cell viability was measured by MTT assay. Results are shown as the mean ± SD of triplicate observations from two experiments. LC50 represents lethal concentration of 50% viability compared with the control.

**Supplementary Fig. S2.** **CQ increased CDDP-induced cell death and DNA damage in RMG-1 cells.** **a** RMG-1 cells were treated with CDDP and CQ for 72 h, and cell viability was measured by MTT assay. Results are demonstrated by a bar graph. **b** Apoptotic cell death was measured by ELISA for detecting active caspase-3. RMG-1cells were treated with CDDP (5 µM) and CQ (20 µM) as indicated for 48 h, and cell lysates were used for caspase-3 assay. **c** RMG-1 cells were treated with either CDDP, CQ, or the combination of CQ and CDDP for 24 h and 48 h. Phosphorylation of histone H2AX (γH2AX) was detected by western blot using anti-γH2AX antibody.

Results are shown as the mean ± SD of triplicate observations from three experiments (n=3, **P< 0.01, ***P< 0.001).

**Supplementary Fig. S3.** **The CQ-CDDP combination arrested cell cycle at G2/M.** **a** RMG-1 cells were incubated with each drug or the combination of drugs for 24 h and 48 h. Cells were stained with propidium iodide and cell cycle distribution was measured by flow cytometry. Flow cytometry was performed three times and the representative data are presented. Cell cycle distribution was demonstrated by a bar graph and the statistical data represented for G2/M arrested cell numbers. **b** Activation and expression of cell cycle-related proteins (Cdc2 and cyclin B1) in RMG-1 cells were examined by Western blot analysis using anti-phospho-specific antibody and antibodies recognizing the total proteins. **c** Expression and activation of ATM and ATR were analyzed in RMG-1 cells by Western blot using phospho-specific antibodies and antibodies recognizing both the phosphorylated and unphosphorylated proteins. β-actin served as protein loading control. Western blot analysis was performed at least three times and the representative figure is presented. The amount of phosphorylated ATM normalized by total ATM expression and ATM expression normalized by β-actin were represented by a bar graph in the upper and lower panels, respectively (n=3, mean ± SD, **P< 0.01, ***P< 0.001). ns represents not significant.

**Supplementary Fig. S4. Single treatment of ATM and ATR inhibitors had no effect on G2/M arrest.** Cell cycle distribution was analyzed in A2780-CP20 cells treated with ATM or ATR inhibitors for 24 h and 48 h by flow cytometry. DMSO was used for the control treatment. Flow cytometry was performed three times and representative data are presented. Cell cycle distribution of 48h-incubation is represented by a bar graph (lower panel). ns represents not significant.

**Supplementary Fig. S5. The CQ-CDDP combination and CDDP single treatment induced p21^WAF1/CIP1^ in RMG-1 cells.** Expression of p21^WAF1/CIP1^ was determined by Western blot in RMG-1 cells treated with CDDP, CQ, or CQ-CDDP for 48 h. β-actin served as protein loading control. Western blot analysis was performed at least three times and representative data are presented. Expression of p21^WAF1/CIP1^ normalized by β-actin was represented by a bar graph (n=3, mean ± SD, **P< 0.01, ***P< 0.001).

**Supplementary Fig. S6. The CQ-CDDP combination inhibited Akt and ROS in A2780-CP20 cells.** **a** Activation of Akt was determined in A2780-CP20 cells treated with CDDP, CQ, or CQ-CDDP for 48 h. Phosphorylated Akt and the total Akt expressions were analyzed by western blot. Ratio of phosphorylated Akt and the total Akt was represented by a bar graph (n=3, mean ± SD, **P< 0.01, ***P< 0.001). ns represents not significant. **b** To detect mitochondrial ROS production, cells were incubated with CDDP, CQ, or CQ-CDDP for 48 h and were stained with MitoSox Red mitochondrial superoxide indicator. ROS production was detected under microscope (left panel). DAPI was used for staining the nucleus. Scale bar represents 50μm. Red-stained cells were counted and were represented by a bar graph (right panel). **c** Expression of Nrf2 and Keap1 was assessed by western blot analysis of A2780-CP20 cells treated with each indicated drug for 48 h. The bar graph was generated with data obtained from three experiments (n=3, mean ± SD, **P< 0.01, ***P< 0.001). **d** senescence was detected in A2780 and A2780-CP20 cells treated with CDDP, CQ, or CQ-CDDP for 48 h by senescence-associated β-galactosidase staining. Stained cells were observed under microscope. Scale bar represents 50μm.
