# Supplementary figures and images for: Chloroquine reverses chemoresistance via upregulation of p21WAF1/CIP1 and autophagy inhibition in ovarian cancer
Source: Cell Death Dis. 2020 Dec 4;11(12):1034. doi: 10.1038/s41419-020-03242-x (PMC7718923; doi:10.1038/s41419-020-03242-x)

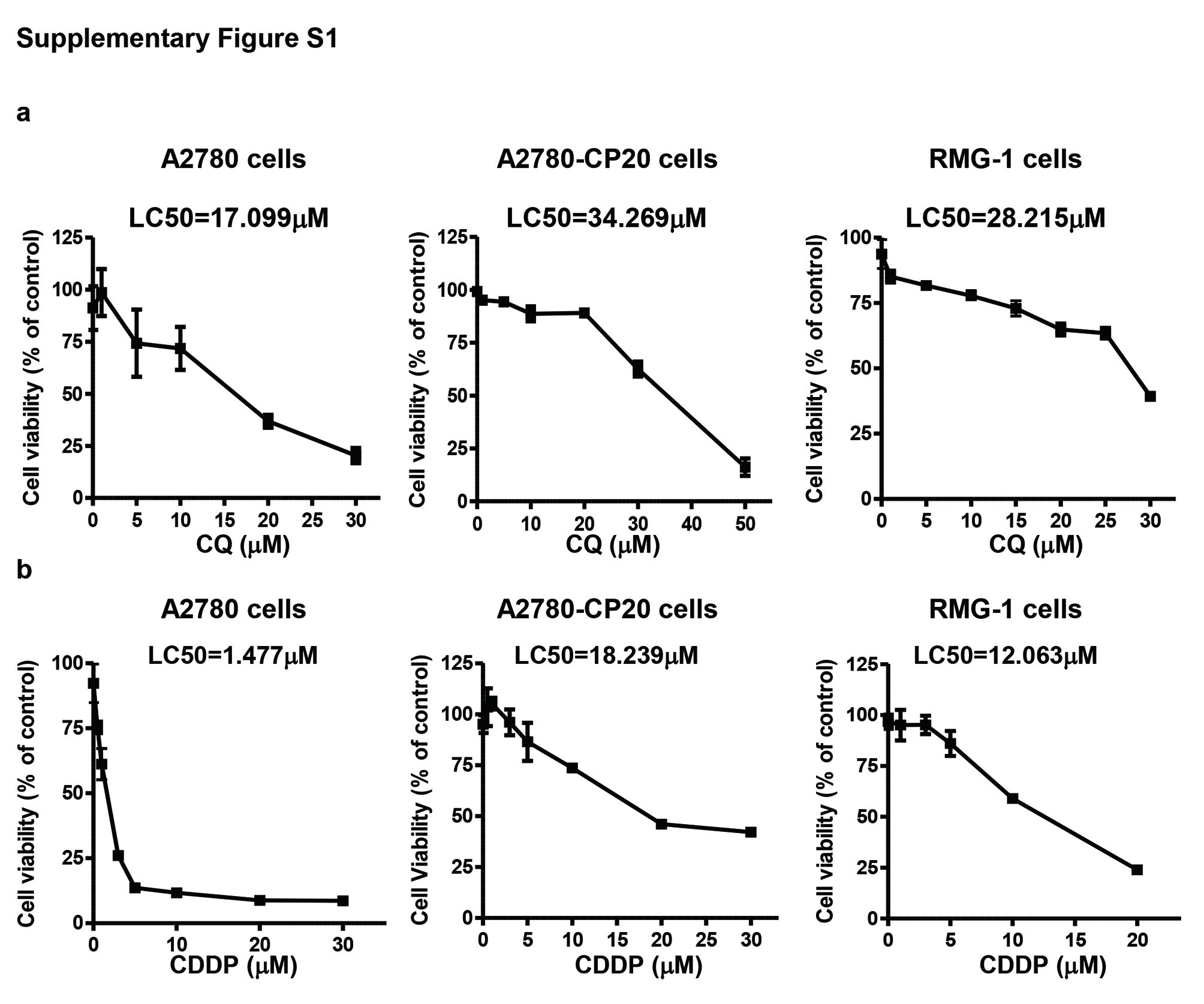

Supplement: Supplementary file 3 — Supplementary Figure S1 [file 41419_2020_3242_MOESM3_ESM.tif]

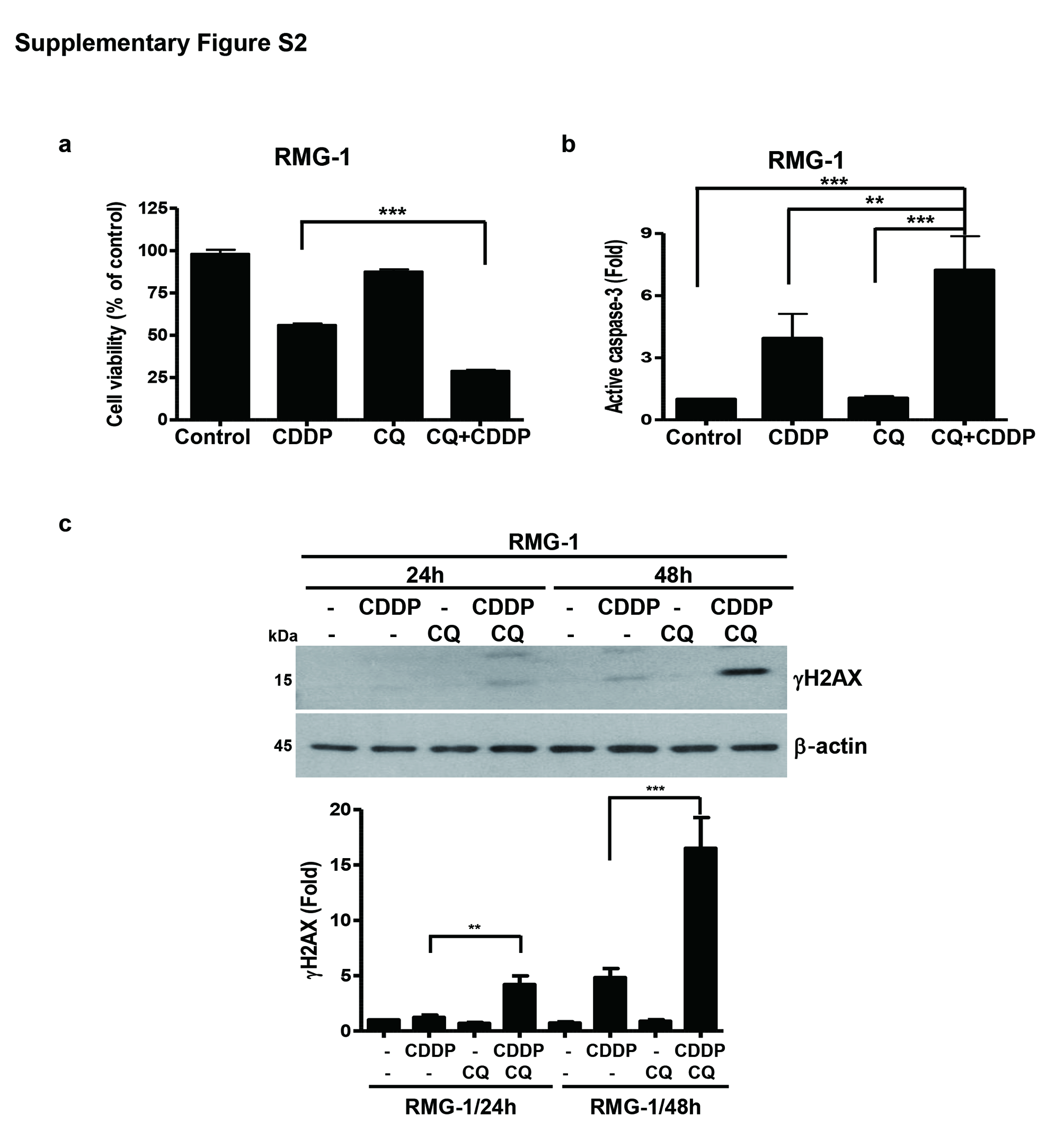

Supplement: Supplementary file 4 — Supplementary Figure S2 [file 41419_2020_3242_MOESM4_ESM.tif]

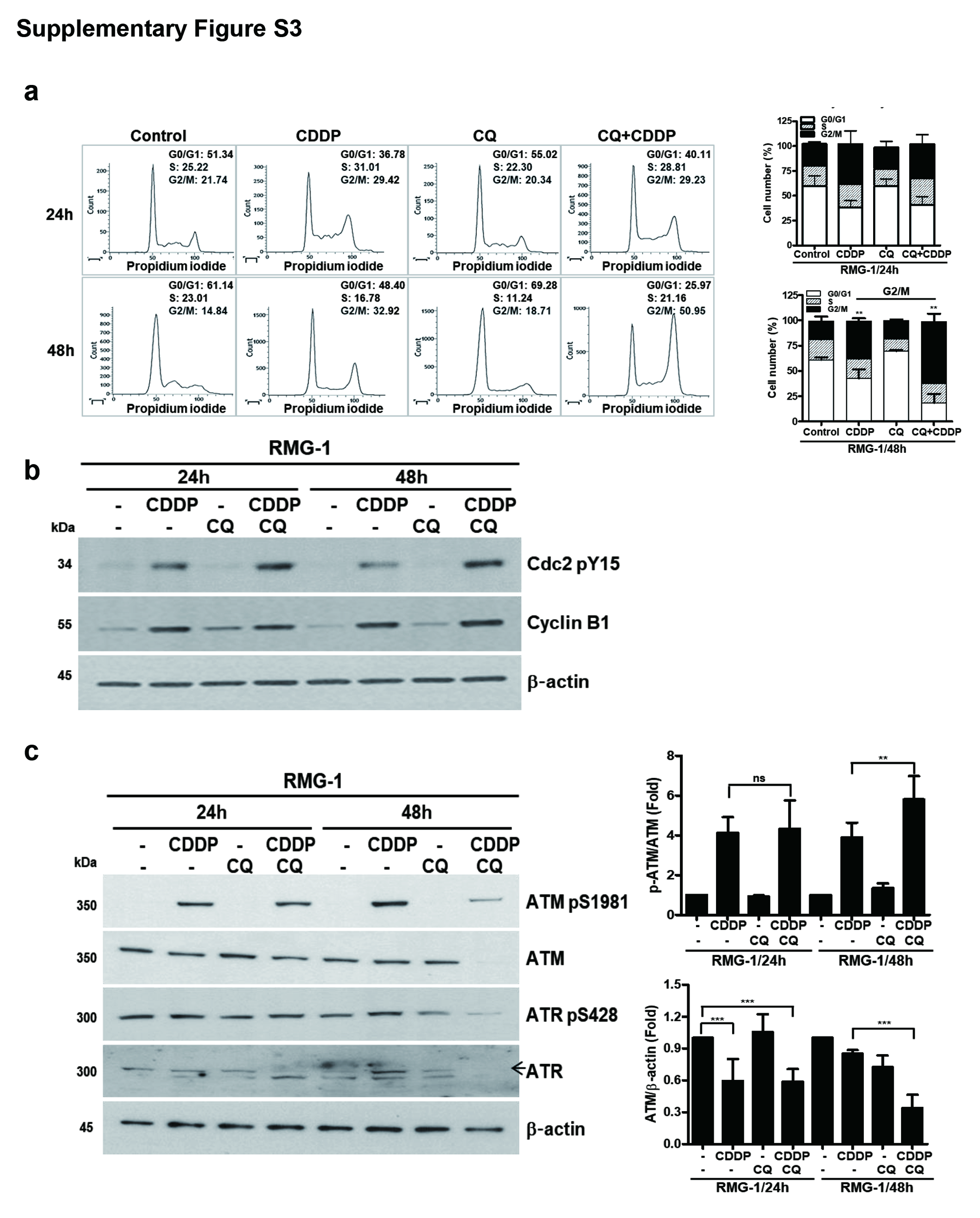

Supplement: Supplementary file 5 — Supplementary Figure S3 [file 41419_2020_3242_MOESM5_ESM.tif]

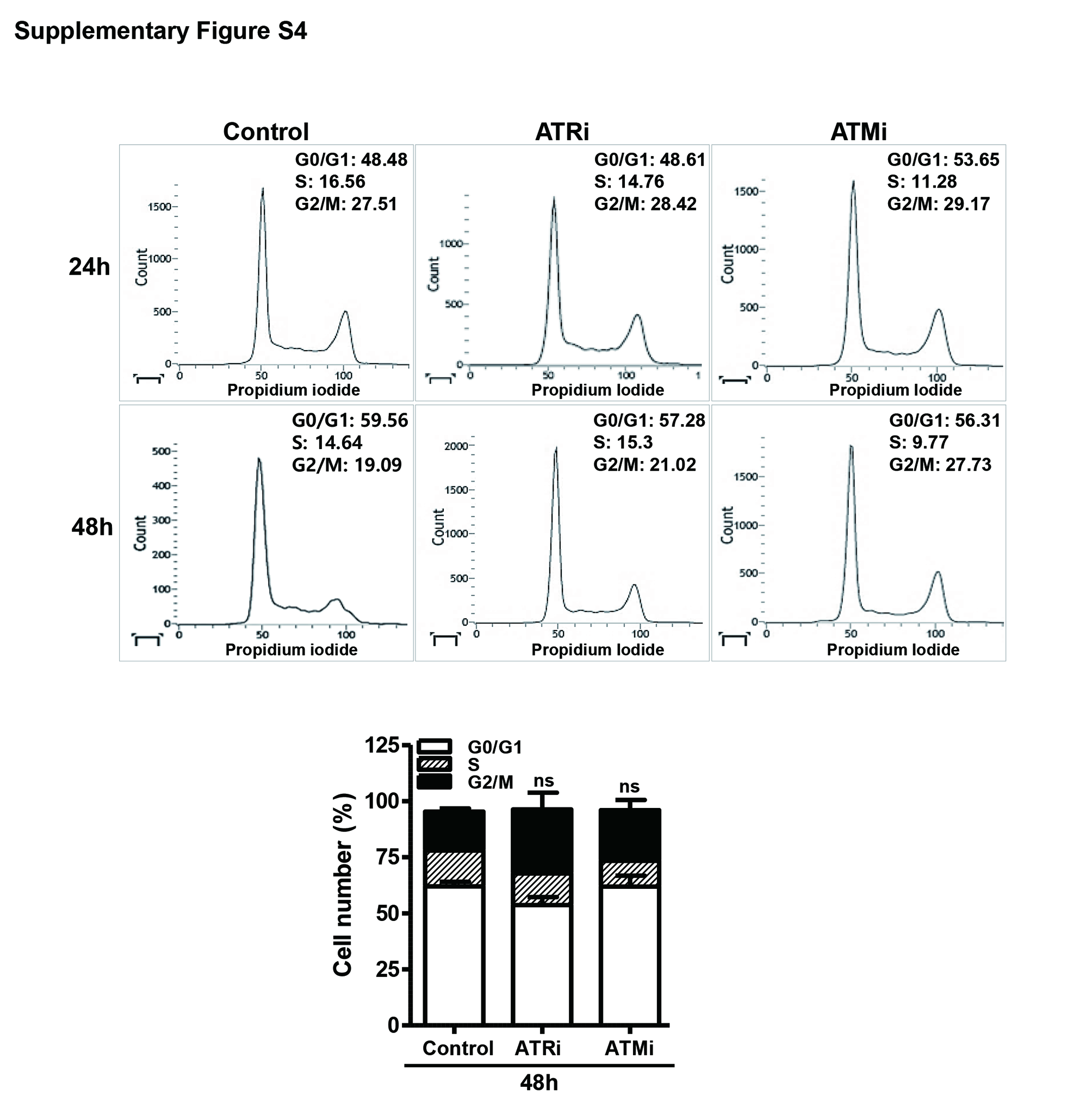

Supplement: Supplementary file 6 — Supplementary Figure S4 [file 41419_2020_3242_MOESM6_ESM.tif]

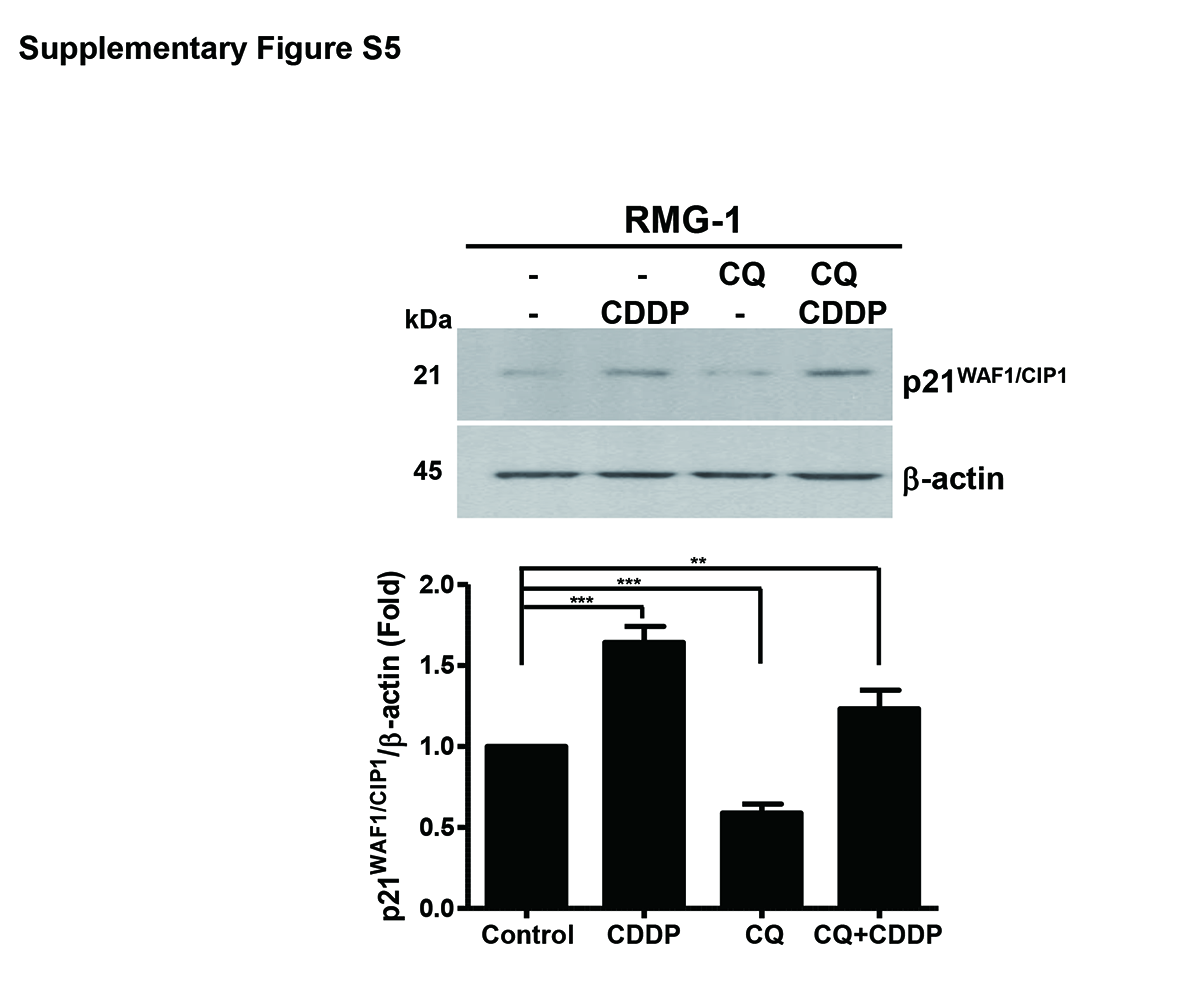

Supplement: Supplementary file 7 — Supplementary Figure S5 [file 41419_2020_3242_MOESM7_ESM.tif]

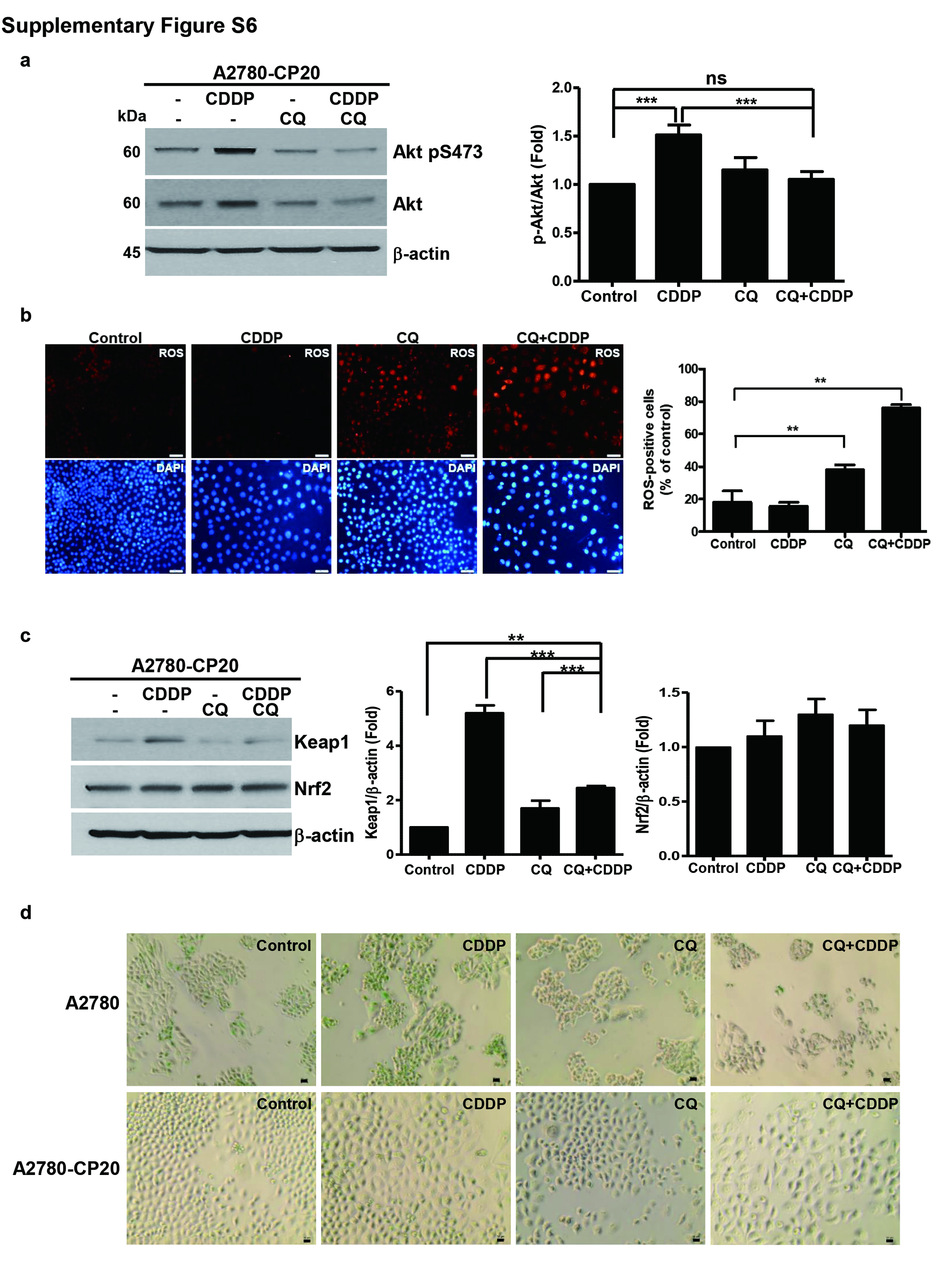

Supplement: Supplementary file 8 — Supplementary Figure S6 [file 41419_2020_3242_MOESM8_ESM.tif]
